# A hybrid modeling framework for generalizable and interpretable predictions of ICU mortality across multiple hospitals

**DOI:** 10.1038/s41598-024-55577-6

**Published:** 2024-03-08

**Authors:** Moein E. Samadi, Jorge Guzman-Maldonado, Kateryna Nikulina, Hedieh Mirzaieazar, Konstantin Sharafutdinov, Sebastian Johannes Fritsch, Andreas Schuppert

**Affiliations:** 1 Institute for Computational Biomedicine, RWTH Aachen University, Aachen, Germany; 2 Department of Intensive Care Medicine, University Hospital RWTH Aachen, Aachen, Germany; 3 Jülich Supercomputing Centre, Forschungszentrum Jülich, Jülich, Germany; 4 Center for Advanced Simulation and Analytics (CASA), Forschungszentrum Jülich, Jülich, Germany

**Keywords:** Machine learning, Interpretability, Generalizability, Hybrid modeling, ICU mortality prediction, ICD codes, Influenza virus, Risk factors, Computational models, Data processing, Machine learning, Predictive medicine, Computer modelling

## Abstract

The development of reliable mortality risk stratification models is an active research area in computational healthcare. Mortality risk stratification provides a standard to assist physicians in evaluating a patient’s condition or prognosis objectively. Particular interest lies in methods that are transparent to clinical interpretation and that retain predictive power once validated across diverse datasets they were not trained on. This study addresses the challenge of consolidating numerous ICD codes for predictive modeling of ICU mortality, employing a hybrid modeling approach that integrates mechanistic, clinical knowledge with mathematical and machine learning models . A tree-structured network connecting independent modules that carry clinical meaning is implemented for interpretability. Our training strategy utilizes graph-theoretic methods for data analysis, aiming to identify the functions of individual black-box modules within the tree-structured network by harnessing solutions from specific max-cut problems. The trained model is then validated on external datasets from different hospitals, demonstrating successful generalization capabilities, particularly in binary-feature datasets where label assessment involves extrapolation.

## Introduction

Hospitalization in the intensive care unit (ICU) is characterized by a variety of disease types and severity; some patients, for instance, might require treatment due to problems with a single organ, while others present failure in many. Through years of training and clinical experience, physicians develop a sense of what a “severe course” looks like, but precisely assessing a patient’s condition at a certain time point is difficult and case-specific. A more standard, quantitative estimation would enable comparative observations, clear communication between multidisciplinary teams, and assist in the initiation of appropriate treatment strategies. As severe courses are associated with high mortality rates, it would also foster more precise prognoses and improve communication with patients’ relatives.

Addressing this challenge, several clinical prediction models and scoring systems have been proposed to serve a variety of critical care applications. These include predicting mortality risk^1^, determining the need for mechanical ventilation^2^, or estimating the length of ICU stay^3^. These models and systems usually include two components: a risk score, which is obtained based on underlying data, and a predictive model, which assigns a patient to a specific risk group^4^. Some are calculated from data collected upon admission^5,6^; others are repeatedly computed every day and can be used to assess the severity of a patient dynamically^7,8^. More recently, with the advent of machine learning and deep learning models, clinical prediction models and risk scoring systems are evolving to sophisticated models incorporating not only routinely measured parameters, but heterogeneous types of data, such as medical notes^9^ or radiological images^10,11^. Several prognostic models and scores have been developed for COVID-19 as well^10,12,13^.

Accounting for comorbidities (multiple, coexisting diseases) in machine learning-based clinical models is often based on annotations that frequently appear in ICU data repositories in the form of codes from the International Classification of Diseases (ICD); healthcare personnel would assign them to a patient verbatim or as abstract diagnoses and update them throughout the patient’s whole stay. Hospitals rely on ICD codes for a variety of purposes such as statistics, billing, and claims reimbursement^14^; research, in fact, comprises a secondary utilization. Numerous applications exist, however, such as retrospective diagnosis studies^15^, identification of patient subpopulations based on distance metrics^16^, or more generally as features in machine learning models that integrate diverse types of data^17^.

Developing clinical predictive models for ICU patients using ICD codes is a significant challenge for machine learning models. One of the primary obstacles lies in the vast number of unique codes within the ICD coding system, where each code represents a specific diagnosis or procedure. Even subsets of these codes can be viewed as high-dimensional data, especially when taking into account the limited amounts of ICU data usually available to researchers. This circumstance brings up issues related to learning in high-dimensional spaces, including the curse of dimensionality^18,19^. Additionally, ICD codes are represented as binary data, implying that any prediction made for unseen data is essentially an extrapolation^20,21^. This can potentially lead to inaccurate, inconsistent, and unreliable predictions, particularly in situations where a patient’s condition is rare or unique.

In light of these complexities, two characteristics become particularly desirable:

- Interpretability: Machine learning approaches are often referred to as “black-box models”; they are powerful at retrieving patterns and making predictions from data, but they do not provide information on the underlying mechanisms of the system they study nor on how their output is calculated. The field of interpretable AI aims to develop machine learning models that are easy to understand^22^; simple examples are decision trees or linear regression, but more elaborated structures incorporating deep neural networks are available as well^23,24^. The discussion is especially relevant in high-stakes scenarios^22^; in healthcare, physicians need to be able to understand what the model is capturing in order to make better-informed decisions^25^. A notable example is the Sequential Organ Failure Assessment (SOFA) score; while initially designed to assess organ dysfunction in sepsis patients^7^, it was later noticed that repeated measurements correlated with mortality^8^, it even became essential in the definition of sepsis in the frame of the Sepsis-3 consensus^26^. Other examples of interpretable clinical predictive models and scoring systems in medicine are, to name a few, for mortality prediction in patients with heart failure^27^, or mortality in general^28^.
- Generalizability: The lack of consistent performance of machine learning models in the clinical setting when tested across data sets of different origins, e.g. from different countries or hospitals, has become an apparent problem^15,25,29–32^. It is related (but not restricted) to difficulties in data acquisition and quality, ambiguous definitions of patient subpopulations or desired clinical outcomes, insufficient demographic representation and other data biases, clinical practice variations in time and geographical locations, and so on^25,31^. Different methods have been suggested to account for this issue^33,34^, as well as guidelines like the TRIPOD (Transparent Reporting of a multivariable prediction model for Individual Prognosis or Diagnosis) statement^35^ or the PROBAST tool^36^.

Incorporating a priori knowledge of the system into the learning task could offer a promising solution to address the concerns mentioned above. One effective approach to achieving this integration is through the utilization of structured hybrid models^37^. These models combine mechanistic and machine learning components in a structured manner, such as organizing them into a tree-shaped network of sub-networks. Each sub-network serves as a distinct machine learning model, rather than using a conventional fully connected neural network. Mechanistic models use mathematical equations based on well-known natural laws, be they physical, chemical, biological, etc., to describe a system. Purely mechanistic approaches tend to not be implemented on real-world clinical problems because the necessary prior knowledge is often incomplete. Machine learning, on the other hand, uses measurements or observations to build a model. Complications, for instance, are the exponential growth of data demand with respect to the number of variables (the curse of dimensionality) and the impossibility of predicting outside of the domain of the observations (lack of extrapolation)^38^.

Hybrid modeling aims to overcome the limitations of each approach. The structure of the hybrid models makes them more interpretable, and if appropriate, helps them to achieve better estimations beyond their training domain^39,40^, even with limited data^38^. They have been particularly successful in the chemical industry^41^ and there are a few examples of applications in the clinical setting^25,32,38^ and systems biology^42^.

In this paper, we introduce a novel structured hybrid model that uses ICD codes for mortality prediction of mechanically ventilated, influenza and pneumonia patients in the ICU. Using graph theoretic approaches, we design a tree-structured network connecting independent modules, each carrying clinical meaning, that leads to an accurate and interpretable mortality prediction framework. We further conduct an external validation study of our model on data sets from different healthcare settings, reporting generalizability and consistent interpretations of mortality causes. Our framework represents a step forward in the development of interpretable and generalizable predictive models in medicine and has the potential to assist physicians in the assessment of critically ill patients and decision-making.

The paper is organized as follows: in “Data resources” section, we introduce the study sample. In “Results” section, we present our designed tree-structured network and describe how its structure integrates underlying medical knowledge for ICU mortality prediction. This is followed by an external validation study and then the consistency check of the mortality interpretations across datasets from different hospitals, concluding with diagnosis-based interpretations. In “Discussion” section, we introduce potential applications of our findings and summarize the main contributions of the paper, its limitations, and areas for future work. In “Methods” section, we describe the model derivation and the numerical and mathematical formulation of our novel graph theory-based learning algorithm.

## Data resources

Data from five German hospitals (hereinafter referred to as Derivation Hospital and Validation Hospitals 1–4) were retrospectively sourced and thoroughly depersonalized from ICU patients involved in the project titled “Algorithmic surveillance of ICU patients with acute respiratory distress syndrome” (ASIC)^43^. This project is an integral part of the SMITH consortium^44^, a body within the German Medical Informatics Initiative.

The patient selection criteria stipulated that the participants must be aged 18 years or over and have experienced invasive mechanical ventilation for a minimum cumulative duration of 24 h. Notably, there were no established exclusion criteria for the study. The data acquired from each patient encompassed routinely charted ICU parameters amassed throughout the entirety of their ICU stay, biometric data, and relevant ICD-10 codes. The ICD codes contained within the dataset encompass both those assigned at the time of admission and during the hospitalization period.

Table 1 outlines the characteristics, health and demographics, and prevalence of specific conditions of the study sample gathered from five German hospitals. The studied sample consisted of severely ill patients who were diagnosed with influenza and pneumonia and required invasive mechanical ventilation at least once throughout their ICU stay. Data from one of the five hospitals, which we refer to as the Derivation Hospital, was employed to train our model, thereby refining its reproducibility for new samples within the same target population. On the other hand, data from the remaining hospitals, termed Validation Hospitals, were utilized to evaluate the model’s performance beyond the derivation sample, thereby assessing the model’s generalizability.

**Table 1:** Characteristics of the studied patient cohorts from five German hospitals.

Cohort characteristics	Derivation Hospital	Validation Hospital 1	*P* value	Validation Hospital 2	*P* value	Validation Hospital 3	*P* value	Validation Hospital 4	*P* value
No. patients	1391 (100%)	254 (100%)	–	558 (100%)	–	948 (100%)	–	2171 (100%)	–
Mortality	439 (31.6%)	78 (30.7%)	0.78	358 (64.2%)	< 0.05	219 (23.1%)	< 0.05	536 (24.7%)	< 0.05
Days of ICU stay	22.5 (19.9)	21.9 (8.2)	< 0.05	21.4 (20.2)	< 0.05	22.9 (21.7)	< 0.05	15.9 (17.9)	< 0.05
Health and demographics									
Age, years	66.6 (12.7)	67.9 (14.5)	0.12	68.1 (13.7)	0.06	66.7 (12.8)	0.19	68.2 (14.0)	0.93
BMI, kg/m^2^	28.9 (6.9)	29.3 (7.8)	< 0.05	28.9 (7.2)	0.09	28.7 (7.4)	< 0.05	27.5 (6.9)	< 0.05
Female gender	457 (32.9%)	99 (39.0%)	< 0.05	188 (33.7%)	0.72	313 (33.0%)	0.93	832 (38.3%)	< 0.05
Diabetes mellitus	470 (33.8%)	92 (36.1%)	0.45	181 (32.4%)	0.56	261 (27.5%)	< 0.05	419 (19.3%)	< 0.05
Thoracic trauma	115 (8.3%)	12 (4.7%)	< 0.05	53 (9.5%)	0.38	99 (10.4%)	0.07	102 (4.7%)	< 0.05
ICD codes									
Renal failure (N17–N19)	777 (55.9%)	118 (46.5%)	< 0.05	445 (79.8%)	< 0.05	406 (42.8%)	< 0.05	1,153 (53.1%)	0.11
Sepsis (A41)	1006 (72.3%)	101 (39.8%)	< 0.05	464 (83.2%)	< 0.05	513 (54.1%)	< 0.05	759 (35.0%)	< 0.05
Diseases of liver (K70–K77)	281 (20.2%)	51 (20.1%)	0.89	307 (55.0%)	< 0.05	59 (6.2%)	< 0.05	320 (14.7%)	< 0.05
Other bacterial diseases (A30–A49)	826 (59.4%)	127 (50.0%)	< 0.05	450 (80.7%)	< 0.05	499 (52.6%)	< 0.05	829 (38.2%)	< 0.05
Diseases of the genitourinary system (N00–N99)	991 (71.2%)	159 (62.6%)	< 0.05	500 (89.6%)	< 0.05	552 (58.2%)	< 0.05	1,409 (64.9%)	< 0.05
Mycoses (B35–B49)	230 (16.5%)	81 (31.9%)	< 0.05	80 (14.3%)	0.22	214 (22.6%)	< 0.05	520 (24.0%)	< 0.05
Liver failure (K72)	173 (12.4%)	33 (13.0%)	0.75	287 (51.4%)	< 0.05	35 (3.7%)	< 0.05	237 (10.9%)	0.27
Mental and behavioural disorders (F10–F19)	710 (51.0%)	98 (38.6%)	< 0.05	198 (35.5%)	< 0.05	604 (63.7%)	< 0.05	1,039 (47.9%)	0.10
Organic, including symptomatic, mental disorders (F00–F09)	548 (39.4%)	66 (26.0%)	< 0.05	104 (18.6%)	< 0.05	488 (51.5%)	< 0.05	723 (33.3%)	< 0.05
Polyneuropathies and disorders of the peripheral nervous system (G60–G64)	188 (13.5%)	36 (14.2%)	0.98	82 (14.7%)	0.40	151 (15.9%)	0.09	173 (8.0%)	< 0.05
ARDS (J80)	382 (27.5%)	77 (30.3%)	0.56	343 (61.5%)	< 0.05	176 (18.6%)	< 0.05	352 (16.2%)	< 0.05
Respiratory diseases principally affecting the interstitium (J81–J84)	397 (28.5%)	82 (32.3%)	0.32	345 (61.8%)	< 0.05	182 (19.2%)	< 0.05	452 (20.8%)	< 0.05

Variable distributions are reported as $n (\%)$ for categorical variables and *mean*(*SD*) for continuous variables. *P* values are obtained using two-sample proportion z-tests and the Mann–Whitney U test, indicating significant differences from the Derivation Hospital.

The cohort characteristics described in Table 1 state that the Derivation Hospital hosted 1391 patients with a mortality rate of 31.6%. The average duration of ICU stay in this hospital was 22.5 days. Similar information for the four validation hospitals is also provided, with patient numbers ranging from 254 to 2171, mortality rates between 23.1 and 64.2%, and average ICU stays from 15.9 to 22.9 days. The health and demographic information presented in Table 1 discloses that the average age of patients was fairly similar across all hospitals, fluctuating around 66–68 years. The average Body Mass Index (BMI) was around 28–29 in all hospitals except Validation Hospital 4, which had a slightly lower average BMI of 27.5. The proportion of female patients in all hospitals varied slightly between 27.8 and 35.8%.

The table also details the presence of several clinical conditions diagnosed per the ICD coding system. While the most recent version of the ICD codes, namely ICD-11, was introduced in January 2002, its previous version is the most used in practice. ICD-10 is an alphanumeric system that uses a hierarchical structure; the first 3 digits represent common traits and each subsequent character, up to seven, provides further specification^45^. For each of the codes, disease categories (ICD chapters) and high-level clinical conditions (ICD blocks) can be extracted. For instance, codes in the range A00–A09 can be mapped first onto the ICD chapter “Certain infectious and parasitic diseases” and then onto an ICD block “Intestinal infectious diseases”. Implementation in machine learning usually involves shortlisting the codes in order to delimit a specific condition of interest, which requires the assistance of medical experts^46^. Table 1 also gives prevalences of specific clinical conditions, including renal failure, sepsis, liver diseases, and others that were used to design our hybrid model for ICU mortality prediction. In some cases, these health conditions exhibit considerable variation across the five hospitals, in terms of the proportion of patients diagnosed with them. For instance, the occurrence of renal failure (N17–N19) ranged from 42.8% in Validation Hospital 3–79.8% in Validation Hospital 2.

**Figure 1:**
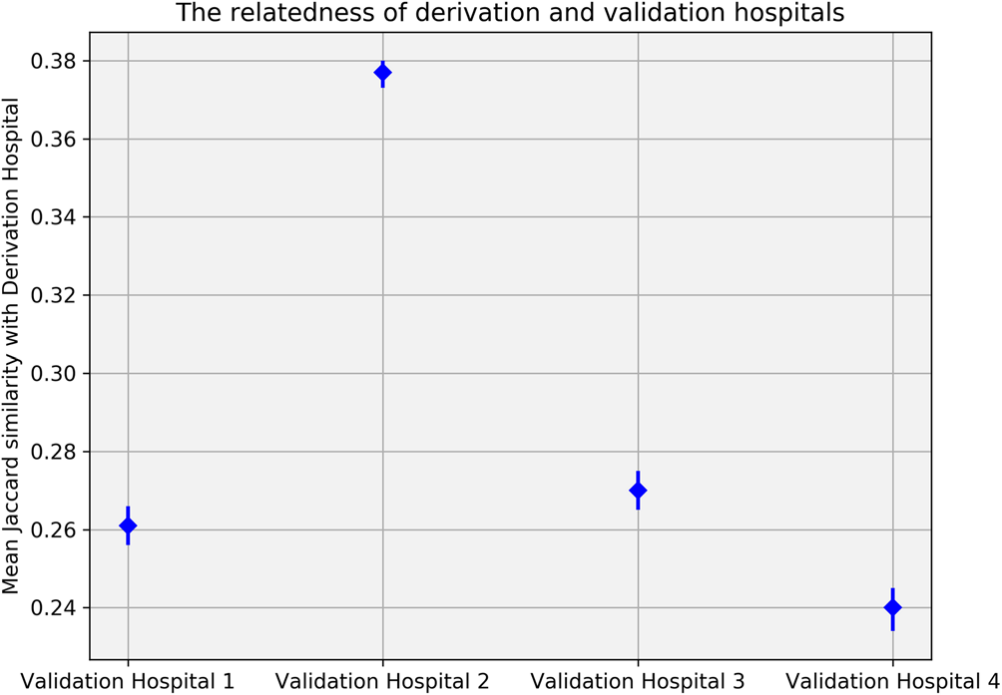
Average and the 95% confidence interval of the Jaccard similarity measures between data samples from a validation hospital and the Derivation Hospital, emphasizing the degree of relatedness between the Derivation Hospital and four validation hospitals.

Figure 1 depicts the extent of relatedness between the data from the Derivation Hospital and the datasets from the four validation hospitals. The figure illustrates the mean and the 95% confidence interval of the Jaccard similarity between all data samples in a validation hospital and all data samples in the Derivation Hospital, please refer to Supplementary Information file under the “Jaccard similarity” section for more details. Through the analysis of results procured via the Jaccard similarity measure, one can easily comprehend the degree of similarity or dissimilarity in the case mix across these hospitals. This invaluable data augments our capability to interpret the results of external validation studies, facilitating our understanding between the reproducibility and the generalizability of our developed model more effectively^47^.

A thorough quantitative bias analysis was performed on the datasets using clinical features presented in Table 1. We utilized Mann–Whitney U tests, a non-parametric statistical test from the SciPy library^48^, to examine continuous variables like age, BMI, and ICU length of stay, revealing any significant differences between the Derivation and Validation Hospitals. Additionally, binary features such as female gender, ICD codes, and mortality were subject to examination via proportions z-tests with the statsmodels library^49^. The resulting *P* values from both Mann–Whitney U tests and proportions z-tests are summarized in Table 1. We highlighted significant differences with *P* values < 0.05, providing a quantitative approach to detect potential biases in the datasets. These analyses, crucial for ensuring the model’s generalizability, are visually presented in the Supplementary Information file under the “Quantitative bias analysis” section.

## Results

### Structured hybrid model development using medical knowledge

The model in this study incorporates medical knowledge related to mortality causes of the critically ill, influenza and pneumonia patients in the ICU. The model derivation and the details of the training strategy are discussed in “Methods” section.

**Figure 2:**
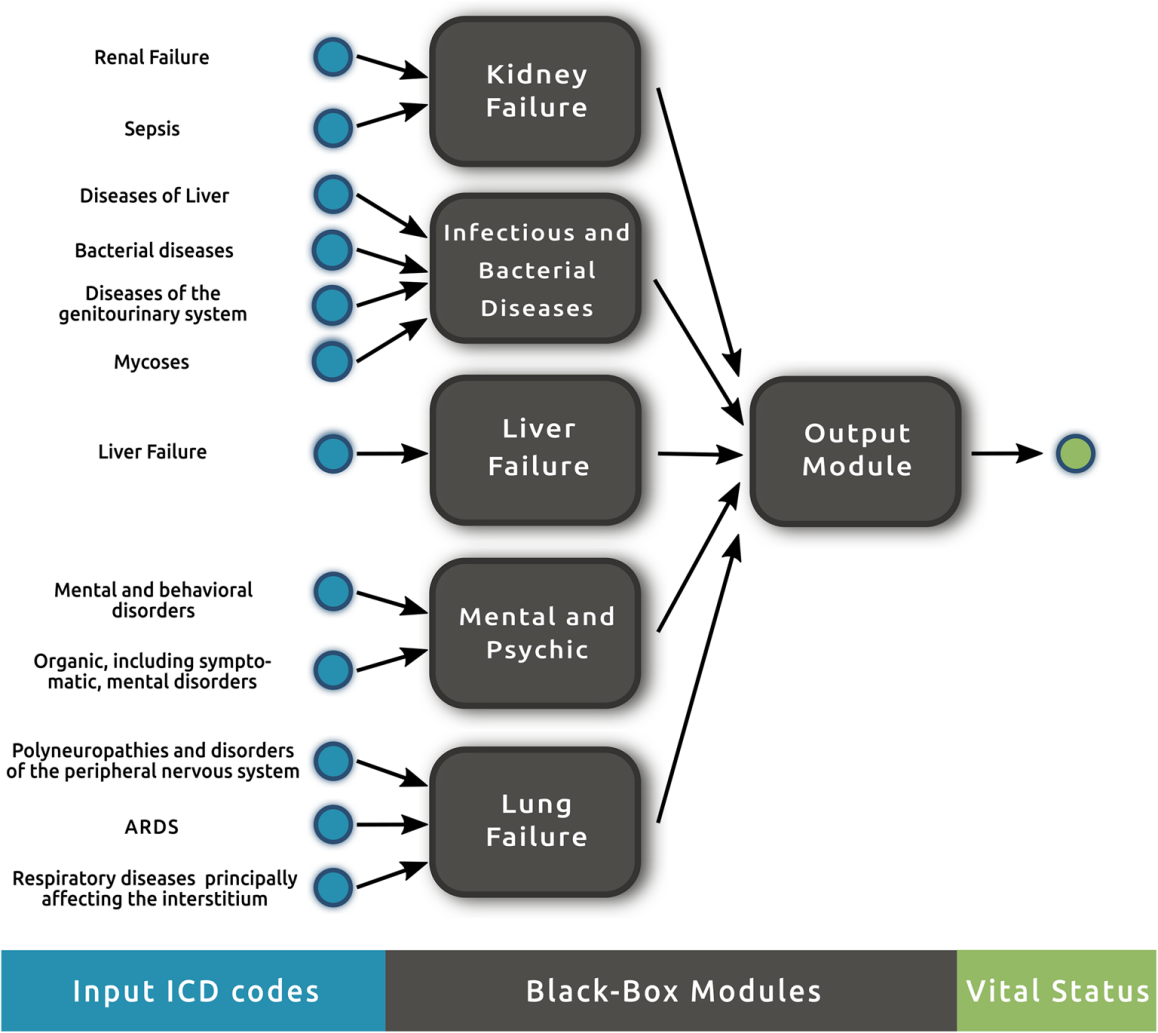
Proposed structured hybrid model for mortality risk stratification of critically ill, influenza and pneumonia patients in the ICU. The model consists of five modules: kidney failure, infectious and bacterial diseases, liver failure, mental and psychic, and lung failure; with their corresponding input features. The output module combines the precomputations of these modules to determine the overall mortality risk of a patient.

A tree-structured network consisting of five independent black-box modules converging into a final, output module is used to compute the mortality risk of a patient in the ICU. Each of the black-box modules in the first layer of the network represents a specific sub-process, taking a subset of the features as input and producing a precomputation of the mortality risk; see Fig. 2. Each first-layer module captures a distinct aspect of mortality causes among critically ill ICU patients. Notably, certain potential causes of mortality, such as heart failure, have been excluded primarily due to their limited discriminative value in the study’s data. For more details, please refer to the Supplementary Information file under the “Heart failure module” section. The final output module then combines the precomputations to predict the vital status of a patient.

- Kidney failure module: This module focuses on the impact of sepsis-associated acute kidney injury (AKI) on patient mortality. Sepsis is a common complication in critically ill patients and can lead to AKI, which is related to increased mortality rates^50–52^. Hence, the Kidney Failure module is designed to study the interplay between “Sepsis” and “Renal failure” and to separately measure the effect of sepsis-associated AKI. By doing so, it assists in the identification of AKI patients at higher risk of mortality.
- Infectious and bacterial diseases module: This module connects several factors that have a high impact on mortality prediction, including “Diseases of liver”, “Other bacterial diseases”, “Diseases of the genitourinary system”, and “Mycoses”. Patients in the ICU are often susceptible to infections, which can worsen their clinical outcomes^53–55^; interactions with liver diseases, which are known to be correlated with mortality themselves^56,57^, have also been reported to have an increasing effect^57–60^. This module helps recognize patients at high risk due to infectious and bacterial diseases, allowing clinicians to provide appropriate interventions such as antimicrobial therapy, infection control measures, and supportive care.
- Liver failure module: “Liver failure” is a critical condition that has a significant impact on the fate of patients in the ICU^61,62^. The Liver failure module is designed to distinguish patients with and without it, allowing for separate measurements of its impact on mortality. This module aids clinicians in spotting patients at high risk, so they can provide targeted treatments such as liver support devices, nutritional support, and management of coexisting conditions.
- Mental and psychic module: The module considers two ICD codes, namely “Mental and behavioral disorders” and “Organic, including symptomatic, mental disorders”, as they have been identified as relevant factors contributing to mortality in the ICU. However, while most studies report a positive association^63,64^, our results suggest a negative one. A few studies similarly report no, or negative associations for a few specific patient subpopulations^65,66^. A notable case is that of delirium, part of the second ICD code involved and well known to positively correlate with mortality in general^67^. As the module weights in several types of mental conditions, these results suggest a highly, marked variability in the effect different mental disorders have on patient mortality.
- Lung failure module: We developed a module to analyze conditions related to lung failure, which is crucial in the study of influenza and pneumonia patients^68,69^. The module takes into account factors such as “acute respiratory distress syndrome (ARDS)”, “Polyneuropathies and other disorders of the peripheral nervous system”, and “Other respiratory diseases principally affecting the interstitium”. We included “Polyneuropathies and other disorders of the peripheral nervous system” as an input to this module, as it has been shown to impact lung functionality and contribute to longer ICU stays^70–72^.

The output module determines the overall mortality of a patient. By combining the precomputations of the previous modules, it captures the interplay between different factors that give rise to complex medical conditions, such as multi-organ dysfunction, that could not be captured by individual modules alone. Further, it is designed to be interpretable; understanding how the factors and their interactions contribute to the final mortality risk provides a clearer picture of individual patients, allowing clinicians to make better-informed decisions and tailor treatments.

### External validation and generalizability

In order to evaluate the efficiency and generalizability of our developed hybrid model, we performed an external validation study, leveraging our data resources from five distinct hospitals. First, the data obtained from the Derivation Hospital served as the training set, which allowed us to determine the black-box module functions within the hybrid model, see Fig. 2. This process was integral to refining the model’s reproducibility. Next, we proceeded to assess the model’s performance beyond the derivation sample, using data from the four validation hospitals. This was a critical step in evaluating the model’s generalizability.

As a benchmark, we compared the results of the external validation study against XGBoost^73^, a widely used machine learning model known for its predictive capabilities. We employed 5-fold stratified cross-validation using the data from the Derivation Hospital as a hyper-parameter tuning method for the XGBoost model, see Supplementary Table S5 for more details.

In terms of evaluation and comparison, we used a spectrum of key performance indicators to determine the classification efficacy of our developed hybrid models and the XGBoost. The indicators chosen were accuracy, recall, precision, F1 Score, and receiver operating characteristic area under the curve (ROC AUC). These metrics collectively offer a comprehensive measure of each model’s performance across various aspects, including overall correctness, sensitivity, specificity, harmonic mean of precision and recall, and discriminative ability, respectively.

Based on the provided averages of the classification metrics in Table 2, the XGBoost model shows slightly better performance during the training phase, indicating its reproducibility. Moreover, it also demonstrates slightly better performance for Validation Hospital 2, which notably shares more similarities with the Derivation Hospital compared to the other validation hospitals, see Fig. 1.

In contrast, our hybrid model demonstrates superior performance over the XGBoost model in the remaining validation hospitals (Validation Hospitals 1, 3, and 4), where their similarities with the Derivation Hospital are less pronounced. Remarkably, our hybrid model displayed a gradual reduction in overfitting as compared to the XGBoost approach and achieved superior metrics including accuracy, recall, precision, F1 score, and ROC AUC for the remaining validation samples. These results underline the improved generalizability of our hybrid model, suggesting its effective application on diverse datasets that extend beyond the original derivation sample.

**Table 2:** Comparison of the averages of the classification metrics for the developed hybrid model and the XGBoost approach across different hospitals.

Hospital	Model	Accuracy	Recall	Precision	F1 Score	ROC AUC
Derivation Hospital	XGBoost	0.937	0.815	0.984	0.892	0.930
	Hybrid	0.899	0.784	0.884	0.831	0.921
Validation Hospital 1	XGBoost	0.843	0.756	0.738	0.747	0.842
	Hybrid	0.874	0.821	0.780	0.800	0.863
Validation Hospital 2	XGBoost	0.918	0.964	0.913	0.938	0.933
	Hybrid	0.894	0.930	0.907	0.919	0.933
Validation Hospital 3	XGBoost	0.898	0.744	0.799	0.771	0.852
	Hybrid	0.902	0.753	0.809	0.780	0.881
Validation Hospital 4	XGBoost	0.886	0.802	0.877	0.860	0.864
	Hybrid	0.904	0.826	0.880	0.896	0.892

**Figure 3:**
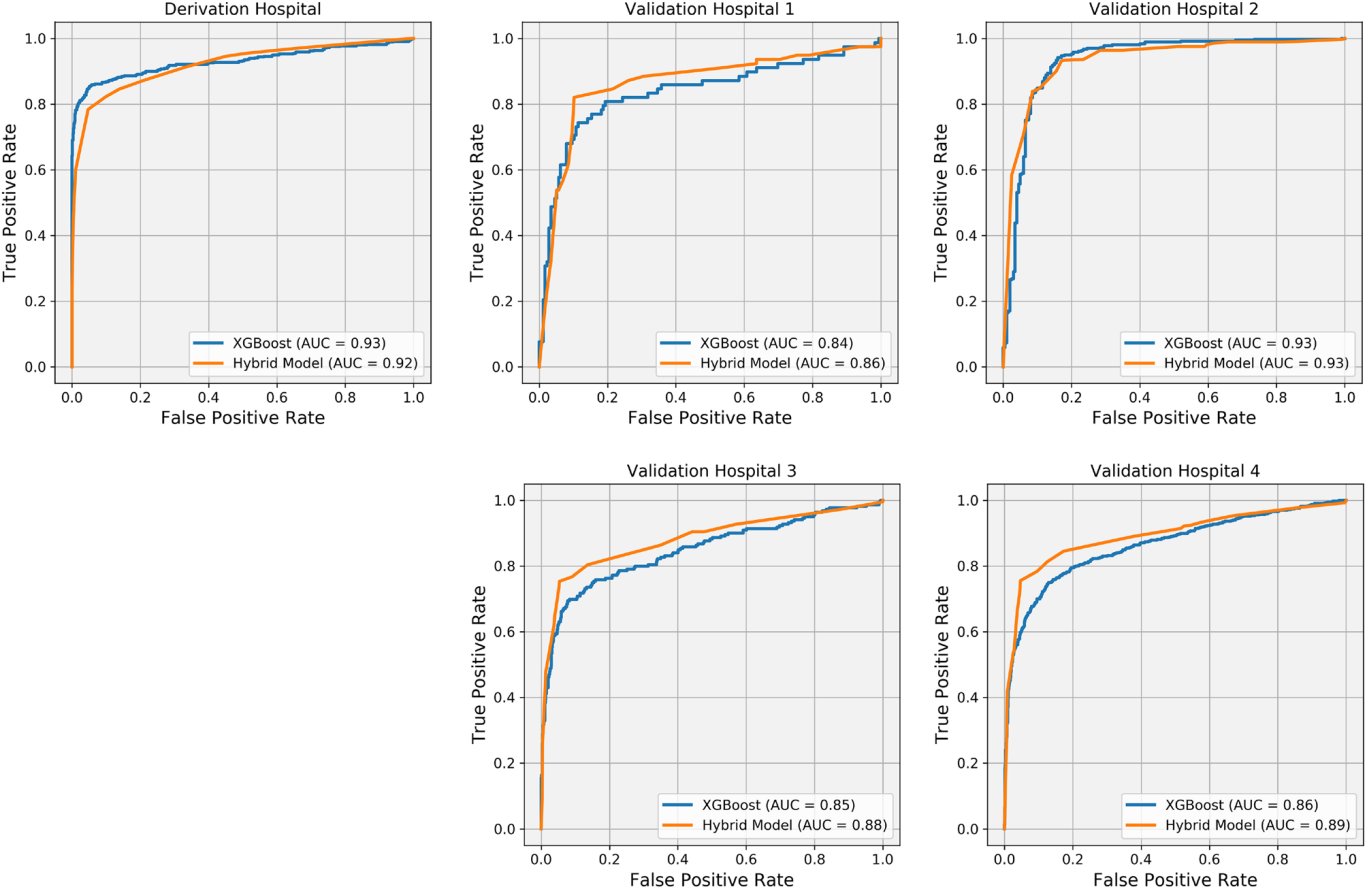
AUC–ROC curves comparing the discriminative ability of our hybrid model and the XGBoost model in distinguishing deceased and alive patients. The hybrid model outperformed XGBoost for Validation hospitals 1, 3, and 4, where their similarities with the Derivation Hospital are less pronounced, highlighting the hybrid model’s generalizability.

Moreover, Fig. 3 depicts the AUC–ROC curves, a visual representation of the discriminative ability of the models. These curves serve as an evaluation metric for the models’ effectiveness in distinguishing between deceased and alive patients. A value of 1 indicates flawless discrimination, while a value of 0.5 signifies random predictions. The x-axis represents the false positive rate, reflecting falsely predicted deceased patients among the actual alive patients, while the y-axis represents the true positive rate, indicating correctly predicted deceased patients among the actual deceased patients. Analyzing the proximity of the curve to the top-left corner provides insights into the models’ classification performance. It was observed that our hybrid model outperforms the XGBoost model specifically for the Validation hospitals 1, 3, and 4, which do not share many similarities with the Derivation Hospital.

### Consistent interpretations of mortality causes

After building and validating our hybrid model, we sought to interpret its predictions. The interpretability of the hybrid model stems both from the structure of the network in Fig. 2 and the learned module functions, each serving as an independent, preliminary mortality estimation for a relevant medical condition. Further details on the approximation of the module functions can be found in “Methods” section. A related concept to interpretability, namely explainability differs mainly in that its goal is to understand an already constructed black box, instead of building a transparent model from the start. Further discussion on the differences and relationships between both concepts can be found in Ref.^74^.

In our study, we employed the use of SHapley Additive exPlanations (SHAP) values, as detailed in Refs.^28,75^, to conduct a comparative analysis of the interpretability between our hybrid model and the XGBoost model. SHAP values provide a unified measure of feature importance in complex machine learning models, augmenting their interpretability. This is achieved by considering both the primary effects of a feature and its interaction effects with other features.

SHAP values are derived from a concept in cooperative game theory known as the Shapley value. This value assigns a payout to each player in a game based on their contribution to the total payout. When we translate this concept into the realm of machine learning model interpretation, the “players” become the input variables or features of the model, and the “game” is the prediction that the model generates. When the model executes a prediction—or in other words, when the “game” is played—each feature, like a player in the game, is assigned a Shapley value. This value, similar to a payout, quantifies the specific feature’s contribution to the final prediction. This analogy serves to enhance our understanding of the impact of individual features on the model’s predictive decisions.

**Figure 4:**
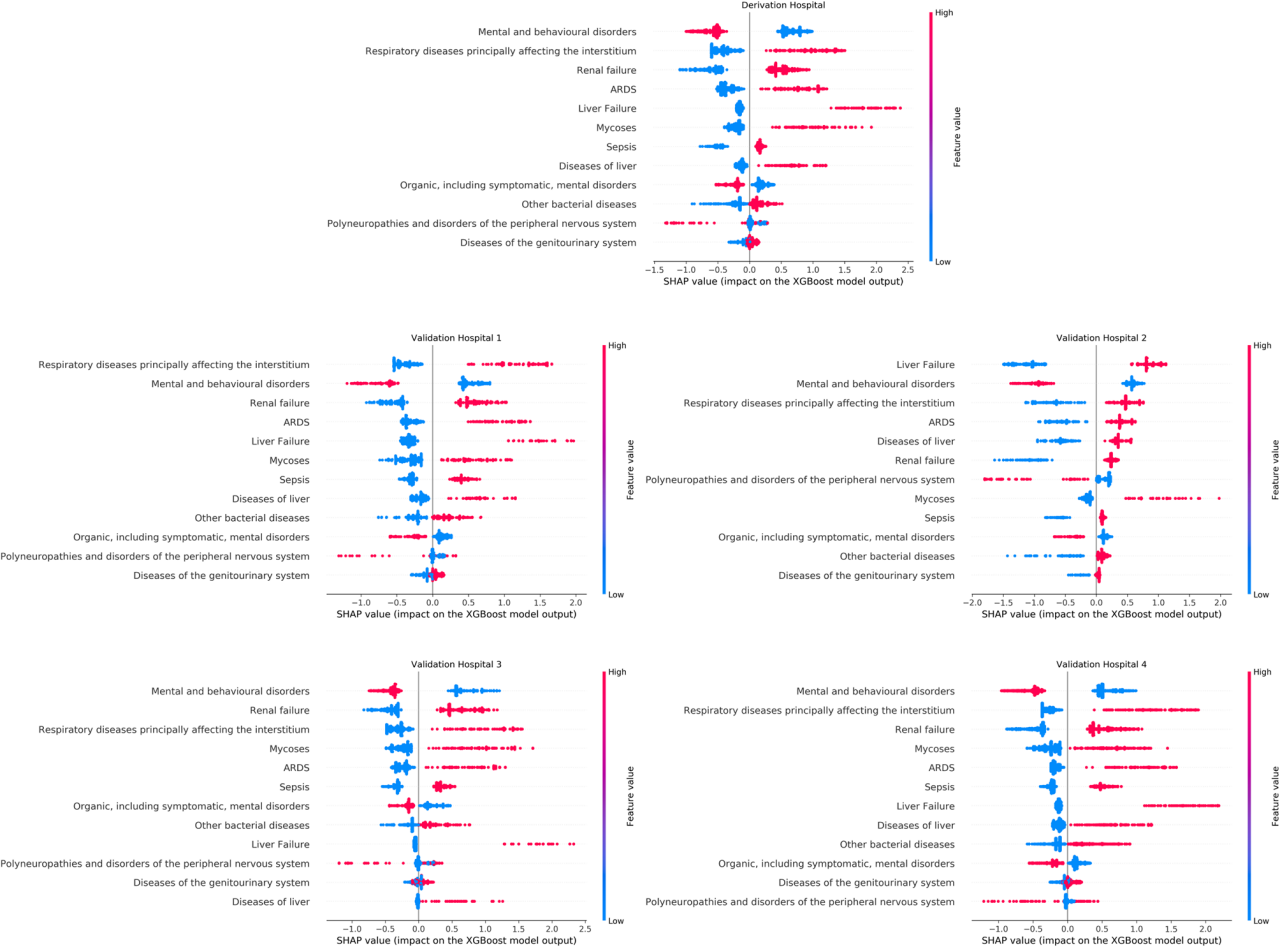
SHAP values distribution for 12 ICD codes in the XGBoost model, used to interpret ICU mortality causes. The figure showcases inconsistency in the feature importance across the five hospitals involved in the study.

In this study and in the case of the XGBoost model, the use of SHAP values provides an insightful understanding of how each input, or the 12 ICD codes, influences the mortality risk. For instance, let’s assume “Sepsis” with a high feature value (here 1 since the feature is binary). If the associated SHAP value is a high positive (or negative) number, it means that the presence of Sepsis plays a significant role in determining the high (or low) mortality risk of a patient. Accordingly, Fig. 4 offers an in-depth examination of the impact of SHAP values associated with the 12 ICD codes we employed in the XGBoost model. The results elucidate how the health conditions diagnosed per the ICD coding system contribute to predicting mortality while demonstrating inconsistency in the related feature importance across the five hospitals involved in the study.

**Figure 5:**
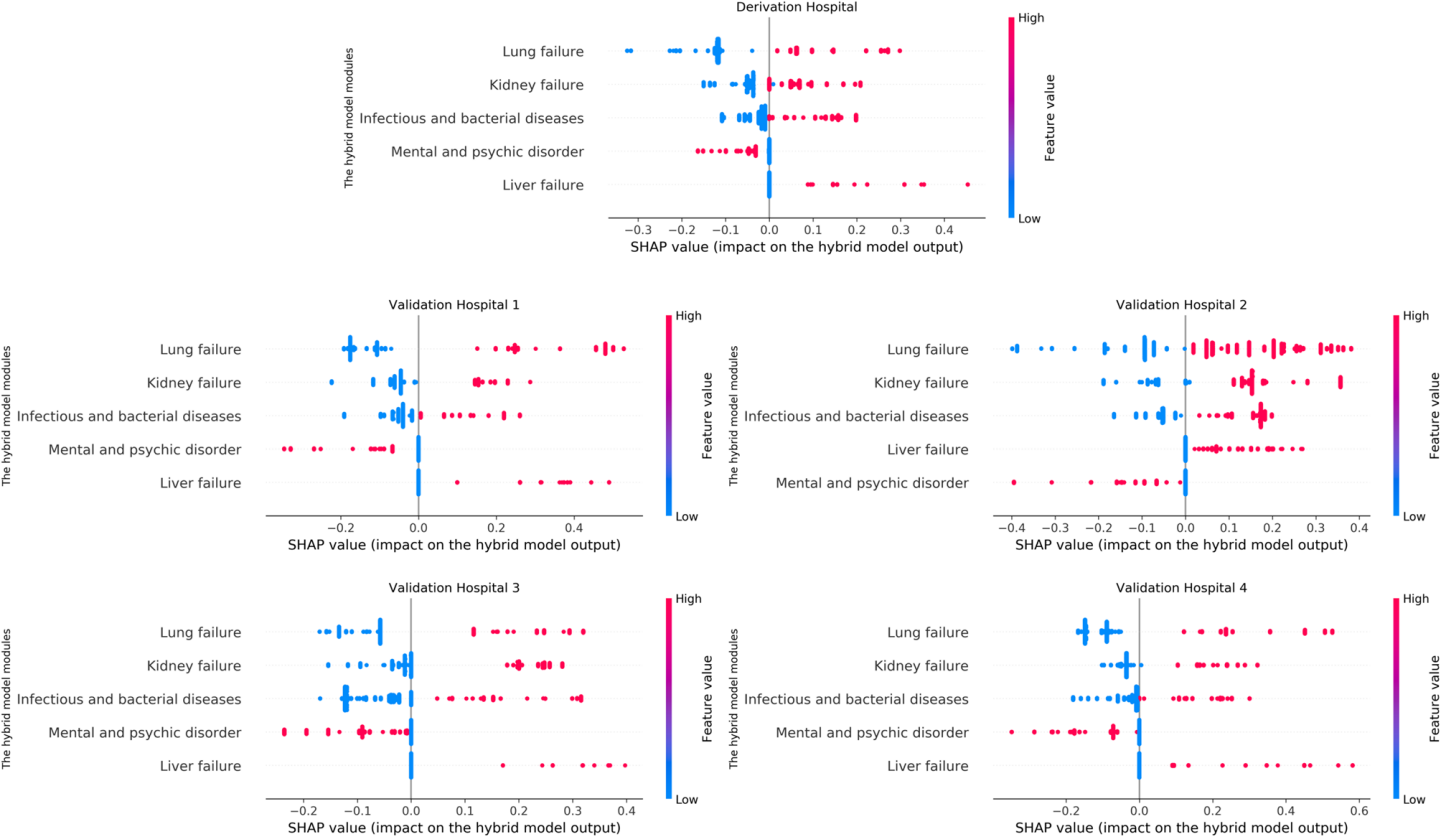
SHAP value distribution for the hybrid model’s black-box modules across five hospitals. The consistency across hospitals showcases the hybrid model’s interpretability, reliability, and stability in mortality prediction across diverse healthcare settings.

Figure 5 illustrates a detailed view of the distribution of SHAP values corresponding to the black-box modules (instead of 12 ICD code inputs) of our hybrid model across the five hospitals under study. For example, consider the “Lung failure” module with a high feature value (here 1 since the outputs of the modules are binary) demonstrated by a red dot. If the associated SHAP value is a high positive (or negative) number, it means that the presence of lung failure, i.e. when the output of the Lung failure module in the hybrid model equals 1, plays a significant role in determining the high (or low) mortality risk of a patient. The results of Fig. 5 showcase the consistency of the distribution of SHAP values and their related feature importance across multiple hospitals reinforcing the reliability and stability of our hybrid model’s interpretations. By focusing on these modules that carry clinical meaning, we not only simplify the interpretability of our model but also enhance the consistency of the interpretations of the causes of mortality across various healthcare settings.

The consistency of the interpretations provided by SHAP values can be quantitatively measured by a statistical test. To implement this, our initial step involved the partitioning of the validation sample-encompassing 3931 patients from the four Validation Hospitals-into randomly sampled 80 validation data subsets. Subsequently, we calculated for each subset the mean of the absolute SHAP values (notated as $\overline{|\text {SHAP value}|}$) for the features that were used to obtain the interpretations of our Hybrid model and the XGBoost model, both of which were trained using data sourced from the Derivation Hospital. Next, we independently tested for statistical differences between the distributions of $\overline{|\text {SHAP value}|}$ related to the features of each model. This statistical analysis facilitated the assessment of the consistency of the interpretations derived from each model.

We used the Friedman test with the Holm post-hoc test, whose null hypothesis $(H_0)$ states that the means of a pair of $\overline{|\text {SHAP value}|}$ distributions resulting from the interpretations of a model (either our hybrid model or the XGBoost model) are the same. Subsequently, the Holm post-hoc test was employed to adjust the *P* values obtained from the multiple comparisons across all features. The choice of these tests was motivated by two main factors. Firstly, the distributions of $\overline{|\text {SHAP value}|}$ did not exhibit properties of a normal distribution, therefore a non-parametric test was required. Secondly, we needed to compare more than two distributions in each case-five, one per network module in the case of the hybrid model; and the five most important ICD codes in discrimination in the case of the XGBoost model. Supplementary Figure S4 online provides further details regarding the decision process and methodology employed in the statistical test using Statistical Tests for Algorithms Comparison (STAC)^76^.

Table 3 presents the test results with a significance level of $\alpha = 0.1$, for the comparison of the $\overline{|\text {SHAP value}|}$ distribution for each pair of the hybrid model modules. In most cases, except for one, the null hypothesis is rejected in favor of the alternative hypothesis, which states that the distribution of $\overline{|\text {SHAP value}|}$ for a pair of hybrid model modules is different. This observation suggests that there is a distinguishable distribution of $\overline{|\text {SHAP value}|}$ for each module of the hybrid model, highlighting robust feature importance and consistent interpretation of our model across the 80 validation data subsets in the validation sample.

**Table 3:** Friedman test results illustrating distribution differences in the mean absolute SHAP values of the hybrid model modules across 80 validation data subsets in the validation sample.

Comparison between $\overline{|\text {SHAP value}|}$ distributions	*z* statistic	Adjusted *P* value	Test result
Lung failure vs. liver failure	11.05000	0.00000	$H_0$ is rejected
Kidney failure vs. liver failure	8.25000	0.00000	$H_0$ is rejected
Liver failure vs. infectious and bacterial diseases	8.00000	0.00000	$H_0$ is rejected
Liver failure vs. mental and psychic disorder	5.95000	0.00000	$H_0$ is rejected
Lung failure vs. mental and psychic disorder	5.10000	0.00000	$H_0$ is rejected
Lung failure vs. infectious and bacterial diseases	3.05000	0.01144	$H_0$ is rejected
Kidney failure vs. lung failure	2.80000	0.02044	$H_0$ is rejected
Kidney failure vs. mental and psychic disorder	2.30000	0.06434	$H_0$ is rejected
Mental and psychic disorder vs. infectious and bacterial diseases	2.05000	0.08073	$H_0$ is rejected
Kidney failure vs. infectious and bacterial diseases	0.25000	0.40259	$H_0$ is accepted

On the other hand, Table 4 presents the test results with a significance level of $\alpha = 0.1$ for the comparison of the $\overline{|\text {SHAP value}|}$ distribution for each pair of the five most important features in discrimination, using the XGBoost model. In half of the cases, the null hypothesis is rejected in favor of the alternative hypothesis. This observation suggests that there is a non-robust feature importance and inconsistent interpretation of the XGBoost model across the 80 validation data subsets in the validation sample.

**Table 4:** Friedman test results illustrating distribution differences in the mean absolute SHAP values of the five most important features in discrimination in the XGBoost model across 80 validation data subsets in the validation sample.

Comparison between $\overline{|\text {SHAP value}|}$ distributions	*z* statistic	Adjusted *P* value	Test result
Mental and behavioral disorders vs. liver failure	6.97500	0.00000	$H_0$ is rejected
Mental and behavioral disorders vs. mycoses	6.90000	0.00000	$H_0$ is rejected
Respiratory diseases principally affecting the interstitium vs. liver failure	6.60000	0.00000	$H_0$ is rejected
Respiratory diseases principally affecting the interstitium vs. mycoses	6.52500	0.00000	$H_0$ is rejected
Mental and behavioral disorders vs. renal failure	5.12500	0.00000	$H_0$ is rejected
Respiratory diseases principally affecting the interstitium vs. renal failure	2.24000	0.18625	$H_0$ is accepted
Liver failure vs. renal failure	1.85000	0.25725	$H_0$ is accepted
Mycoses vs. renal failure	1.77500	0.25725	$H_0$ is accepted
Mental and behavioral disorders vs. respiratory diseases principally affecting the interstitium	0.37500	1.00000	$H_0$ is accepted
Liver failure vs. mycoses	0.07500	1.00000	$H_0$ is accepted

A fluctuation in feature importance rankings could imply inconsistent interpretations of mortality causes across the different validation data subsets. Tables 5 and 6 showcase the ranking of feature importance determined by the average ranks of the absolute value of the SHAP value of each feature across all 80 validation data subsets for our hybrid models and the XGBoost model, respectively. As illustrated in Table 5, the average feature ranking in the hybrid model is well-separated, thereby demonstrating consistency in the interpretations of mortality causes across the various validation data subsets. On the contrary, the average feature ranking in the XGBoost model presented in Table 6 is not well-separated. This inconsistency suggests variable interpretations of mortality causes across the validation data subsets, thereby making it difficult to draw reliable conclusions about the key discriminative features.

**Table 5:** Feature importance ranking for the hybrid model: the well-separated rankings underscore consistent interpretations of mortality causes across the validation data subsets.

Hybrid model modules	Average importance rank
Lung failure	1.4750
Kidney failure	2.1200
Infectious and bacterial diseases	3.0625
Mental and psychic disorder	3.8750
Liver failure	4.6625

**Table 6:** Feature importance ranking for the XGBoost model: the lack of well-separated rankings indicates inconsistent interpretations of mortality causes across the validation data subsets.

Most important features in XGBoost	Average importance rank
Mental and behavioral disorders	2.03125
Respiratory diseases principally affecting the interstitium	2.12500
Renal failure	3.31250
Mycoses	3.75625
Liver failure	3.77500

### Diagnosis-based interpretations

In addition to interpreting causes of mortality through the parameters employed in a predictive model, incorporating diagnosis-based interpretations is essential to enhance the reliability of the model predictions. While the health history of patients and demographic features may not be discriminative enough for inclusion in the model, they still can play a significant role in risk interpretation. This is because they can either exert a misleading influence on the model’s parameters or encompass aspects that cannot be captured by them.

To facilitate diagnosis-based mortality risk interpretations, we incorporated two health conditions, diabetes mellitus and thoracic trauma, as well as two demographic features, age and gender. The focus of these interpretations is to comprehend the influence of health conditions and demographic features on the decisions made by the predictive model.

Table 7 presents the statistical significance of variations in the prevalence of health and demographic features between the false positive (FP) and true positive (TP) cohorts predicted by our hybrid modeling framework. We computed the prevalence of diabetes mellitus, thoracic trauma, and female gender, alongside the average age for each cohort in each hospital. The risk difference was then calculated as the disparity in prevalence, or in the case of age, the average difference. We used two-sample proportion z-tests to assess the statistical significance of differences in the proportions of diabetes mellitus, thoracic trauma, and female gender between the two cohorts for each hospital. The analysis comparing ages between FP and FN cohorts employed the Mann–Whitney U test. The resulting *P* values are presented for each feature, with a critical evaluation of significance levels at 0.05.

**Table 7:** Risk differences and significance in health and demographic factors between FP and TP cohorts by Hospital.

Health and demographic features	Significance	Derivation Hospital	Validation Hospital 1	Validation Hospital 2	Validation Hospital 3	Validation Hospital 4
Diabetes mellitus	Risk difference	0.045	0.108	0.048	0.192	0.009
	*P* value	0.545	0.380	0.564	< 0.05	0.849
Thoracic trauma	Risk difference	− 0.020	− 0.062	− 0.045	− 0.046	− 0.014
	*P* value	0.609	0.276	0.396	0.365	0.565
Female gender	Risk difference	0.079	− 0.191	0.125	− 0.152	0.042
	*P* value	0.295	0.147	0.139	0.070	0.496
Age	Mean difference	0.039	− 2.459	− 2.210	− 1.086	− 0.557
	*P* value	0.983	0.452	0.409	0.671	0.734

Risk differences are calculated by subtracting the prevalence in the FP cohort from that in the TP cohort, and statistical significance is assessed through two-sample proportion z-tests and the Mann–Whitney U test, with the results indicating *P* values < 0.05.

As an illustrative case, Table 7 presents a consistently elevated prevalence of diabetes mellitus in the FP cohort across all hospitals when compared to the TP cohort, with a particularly significant difference observed in Validation Hospital 3. Interpreting this observation from a diagnosis-based perspective suggests that the presence of diabetes mellitus increases the likelihood of FP predictions by the model. One plausible explanation for this observation is that patients with diabetes mellitus may exhibit high-risk factors in the model parameters, leading to an elevated predicted mortality risk. However, with proper management during the treatment phase in the ICU, their outcomes could potentially be more favorable than predicted by the model.

The same analytical approach was employed for the false negative (FN) and true negative (TN) cohorts, with the results summarized in Table 8. When examining the risk difference of thoracic trauma between these cohorts, a higher prevalence of thoracic trauma is observed in the FN group across most hospitals, especially a significant difference in Hospital 3.

One plausible explanation for this finding is that the severe conditions associated with thoracic trauma may not be adequately captured by the predictive model parameters, leading to higher mortality among patients with thoracic trauma that goes unnoticed by the model. Due to the infrequent occurrence of events like thoracic trauma, the feature may not be substantial enough to serve as a discriminative parameter in the predictive model. Hence, incorporating diagnosis-based interpretations in these cases could provide valuable insights. These interpretations could assist physicians in making more informed decisions by leveraging the model’s predictions, potentially enhancing the reliability of the predictive model in clinical settings.

Finally, in terms of demographic features, as shown in Table 8, there is a significant difference in the increased prevalence of Female gender in Validation Hospital 2 and the average age in Derivation Hospital and Validation Hospital 1 within the FN cohort when compared to the TN cohort. Interpreting this observation from a diagnosis-based perspective suggests potential unreliability in the model’s ability to make accurate predictions for cohorts consisting of elderly female patients in this study. This awareness is crucial for physicians in making well-informed and targeted decisions.

**Table 8:** Risk differences and significance in health and demographic factors between FN and TN cohorts by Hospital.

Health and demographic features	Significance	Derivation Hospital	Validation Hospital 1	Validation Hospital 2	Validation Hospital 3	Validation Hospital 4
Diabetes mellitus	Risk difference	− 0.034	0.042	− 0.030	− 0.003	− 0.058
	*P* value	0.511	0.754	0.771	0.957	0.111
Thoracic trauma	Risk difference	0.039	0.051	0.055	0.142	− 0.021
	*P* value	0.205	0.388	0.376	< 0.05	0.292
Female gender	Risk difference	0.077	0.061	0.203	0.026	0.030
	*P* value	0.123	0.648	< 0.05	0.697	0.490
Age	Mean difference	4.832	9.353	0.835	1.747	1.304
	*P* value	< 0.05	< 0.05	0.778	0.415	0.308

Risk differences are calculated by subtracting the prevalence in the FN cohort from that in the TN cohort, and statistical significance is assessed through two-sample proportion z-tests and the Mann–Whitney U test, with the results indicating *P* values < 0.05.

## Discussion

In this paper, we introduce an interpretable and generalizable hybrid model for stratifying mortality risk in influenza and pneumonia patients in the ICU. The main goal is to leverage this model for clinical decision support applications. Our approach involves using a subset of relevant ICD codes describing a patient’s condition taken as (binary) inputs of the whole model. These are then assigned to five different modules, each specifically designed to sub-stratify mortality risk for a distinct medical condition. The design of these modules and the selection of features are rooted in mechanistic, clinical knowledge as well as previous clinical experiences on the adverse events for ICU patients particularly with respect to multiorgan dysfunction. To ascertain the sub-stratification functions for each module, we implemented a graph theory-based learning strategy to be described in “Methods” section. The results produced by these modules are subsequently collated into a final module, which ultimately calculates the patient’s mortality risk.

We would like to highlight a few aspects of our contribution:

First, by utilizing a structured hybrid model as the core of our stratification system, we gain certain advantages that are not feasible in either a purely mechanistic or solely data-driven approach. On the one hand, a purely mechanistic model faces challenges due to the lack of comprehensive knowledge about all factors affecting patient mortality, including the precise nature of their interactions. On the other hand, solely data-driven approaches encounter significant hurdles when using ICD codes for predictions. The abundance of categories in the ICD coding system, each representing a distinct diagnosis or procedure, poses a considerable challenge for data-driven methods. Dealing with subsets of ICD codes in the context of limited clinical data can lead to problems related to learning in high-dimensional spaces, such as the curse of dimensionality^18,19^. Additionally, since the ICD codes are binary, any predictions beyond the scope of the provided training data are considered extrapolations^20,21^, which can lead to inaccurate, uncertain predictions, particularly in cases where a patient’s condition is rare or unique. A formalism based on hybrid models allows us to make use of the existent clinical knowledge at hand, in this case, the design of the modules and the choice of their respective features, to guide and reduce the learning task to smaller black-box models that might not be complex enough to fall into the problems mentioned above.

Second, in this work, we devised a proof-of-concept model leveraging the ICD codes to predict specific outcomes for patients in the ICU. While our model has been developed using ICD codes assigned both on admission and during the patients’ ICU stay, it can indeed be extended to use ICD codes assigned solely at admission, and even to use discretized baseline values of continuous variables to enhance its predictive capabilities.

Third, the tree structure of the hybrid model inherently makes the model interpretable. The final prediction is an aggregation of five sub-stratification, while each is a black box, it is clear which aspect of the health status of a patient they evaluate. The prediction and their aggregations differ for each patient, so it is possible for clinicians to evaluate both the relevance of the modules and their interactions in individual cases.

Fourth, the ability of our hybrid model to provide consistent and robust interpretations across different external hospitals is crucial in medical decision-making. It ensures that the identified risk factors and their impact on mortality risk can be relied upon when assessing patients’ health and planning appropriate interventions. By leveraging the strengths of our hybrid model and comparing its interpretations provided by SHAP values with the XGBoost model, we gain confidence in the reliability and transferability of our approach.

Significantly, Fig. 4 illustrates a degree of inconsistency in the distribution of SHAP values and their related feature importance across multiple hospitals when considering the XGBoost model. This issue can be understood by considering the calculation method of SHAP values, which involves approximating them through permutations of feature values and subsequently generating predictions based on these altered combinations. When using the XGBoost model with 12 ICD codes serving as inputs, there are $2^{12}$ potential feature permutations. KernelSHAP, a tool employed in the SHAP library^28^, undertakes selective sampling from these permutations for each SHAP value approximation. This approach, however, can lead to inconsistent outcomes, a consequence of the large permutation space and the random selection process. Furthermore, while calculating SHAP values in this scenario, we permute features across all possible ICD code configurations. Such a process might introduce permutations that are either unrealistic or violate physical constraints, resulting in unreliable or even detrimental results.

To address the aforementioned challenge, we implemented our hybrid model that structurally leverages preliminary mortality risk assessments generated by the black-box modules in the first layer of the network of Fig. 2. This modification transforms the “players” in the mortality prediction game from ICD codes to the consequential outputs of the relevant black-box modules of the hybrid model, thereby reducing the permutation space for SHAP value calculation from $2^{12}$ to $2^{5}$. Consequently, we observe a notable improvement in the consistency of SHAP values and their associated feature importance across multiple hospitals, as depicted in Fig. 5.

Fifth, the unambiguous and consistent interpretations yielded by our method possess significant potential as a valuable tool for clinicians. For example, the consistent patterns of the SHAP value distribution for the “Mental psychic disorder” and “Liver failure” modules across all hospitals could serve as an essential tool for elucidating mortality causes in ICU patients. More specifically, the positive outcomes (when module output equals 0) of these modules do not contribute significantly to mortality risk. However, the adverse outcomes (also when module output equals 1) of the “Mental psychic disorder” and “Liver failure” modules respectively contribute in a notable negative and positive manner to an increased risk of mortality.

Sixth, in addressing the practical implementation of our model in clinical settings, we emphasize its minimal computational requirements during training, allowing it to run on standard equipment. For instance, the training phase of this study was executed on a GNU/Linux system equipped with an Intel(R) Core(TM) CPU (i7-8565U @ 1.80 GHz). Recognizing potential integration challenges in diverse clinical settings, such as interoperability with healthcare systems, we acknowledge the need for focused research and development. Future efforts will address these challenges to ensure seamless integration into real-world clinical workflows. Moreover, to enhance practical use, we are actively developing a Python package featuring a clear API and comprehensive documentation. The upcoming release aims to enhance accessibility for a broad range of users in healthcare settings.

This study is subject to some limitations that merit exploration in future research. The initial limitation lies in the scope of the framework presented in this paper which is currently confined to binary input data. We are convinced that our methodology can be extended to encompass continuous input data, as the input for any network is invariably defined within a certain precision, allowing for discretization and binarization. In response to this constraint, our future work involves developing a rigorous data binarization procedure prior to the training phase in order to utilize other types of clinical data in the risk stratification.

The second limitation stems from the graph theory-based training strategy used to identify black-box modules. This strategy faces a constraint in computational time when the number of input variables exceeds 6–8. Consequently, this becomes a barrier when attempting to design more complex structured networks, which involve incorporating additional features into the study. To overcome this limitation, future research could focus on developing heuristic approaches for identifying black-box modules or adapting the existing strategy to leverage exponential computational power.

Moreover, increasing the complexity of the designed structured network can impact the consistency of interpretations. Striking a balance between the number of input features and the number of black-box modules in the structured network is essential to achieve consistent interpretations. This aspect warrants further investigation in our learning strategy, leaving room for future work.

The third limitation stems from the inherent constraints of the patient datasets used in this study, recorded within a relatively short timeframe from March 1, 2020, to December 13, 2021. These constraints impede the comprehensive long-term validation of our model, limiting our ability to gain insights into its effectiveness over extended periods.

To finalize, restricting our scope to a subset of relevant factors that are in general agreed on by the medical community might be advantageous in the search for generalizability, instead of basing the selection on the results given by individual data sets. This in turn reduces the identification problem of the data-driven approach from a huge black box to several, in this case five, significantly smaller ones. This bias, however, has its own risks, as other relevant, underappreciated factors might be ignored. It also complicates the discovery of new influential features. Furthermore, while one of the key ideas of this project is to leverage the applicability of a simple data set such as the ICD codes by means of creative modeling, an implementation relying on more detailed, perhaps time-course, information might be of relevance.

## Methods

In this section, we present the development of our hybrid modeling framework designed for the stratification of mortality risk among ICU patients. The “Model” subsection covers the fundamentals of our developed structured hybrid model, followed by an interdisciplinary review on the model’s practicality and interpretability in real ICU settings, along with an overview of our proposed learning strategy. Moving to the “Learning algorithm” subsection, we present the numerical formulation of our function identification strategy, articulated through a presentation of pseudo-code. In the dedicated “Mathematical formulation of the learning strategy” subsection, our focus centers on the mathematical formulations employed for the function identification of each black-box module within the structured hybrid model.

### Model

Our model integrates data patterns with medical knowledge pertaining to mortality causes commonly observed in ICU patients. It consists of a structured network that takes ICD codes as input features and maps them to a mortality probability. In the end, our model presents high accuracy, generalizability and is interpretable.

Figure 2 illustrates the proposed tree-structured network $\textbf{F}: \{0, 1\}^{12} \longmapsto \{0, 1\}$ mapping 12 binary ICD codes to the mortality probability of mechanically ventilated influenza and pneumonia patients. This network comprises two layers: the first layer consists of independent black-box modules, known as first-layer modules, while the second layer contains one output module. Each first-layer module operates independently on a subset of input features and produces a binary output, performing sub-computations for the main classification task. The output module, situated in the second layer, processes the outputs of the first-layer modules to generate the final outcome of the model.

The interpretability of our developed framework in real ICU settings becomes clear when we recognize that the final model outcome is a combination of five sub-stratifications. Each of these sub-stratifications produces a specific sub-outcome, evaluating distinct aspects of a patient’s health status. This aggregation of sub-outcomes closely mirrors established clinical practices, such as the resemblance observed with the SOFA score^7^, which itself aggregates six distinct scores. Despite being generated by a black-box module, practitioners can easily discern which aspect of a patient’s health status each sub-outcome assesses.

The practicality of our developed framework in real ICU settings revolves around the feature selection process, a crucial aspect within the mechanistic modeling part of our hybrid model. From the extensive pool of available ICD codes pertaining to ICU patients, 12 were selected as the most relevant by a combined strategy of a black box predicting model and medical expertise. Initially, a random forest classifier was employed, utilizing all codes as features to predict mortality. Instead of relying solely on a predetermined threshold of feature importance, we integrated domain knowledge from a medical professional. This input helped us not only shortlist the clinically more relevant features but also organize them into groups that reflect clinical interplay in a structured manner. These organized features then serve as inputs for the black-box modules in the first layer of our hybrid model, see Fig. 2.

The primary contribution of this paper, in the modeling part, lies in formulating the identification of individual first-layer modules as specific maximum-cut *(max-cut)* problems. Our strategy involves converting the training data information into a conflict graph for each of the first-layer modules. Given a conflict graph *G*(*V*, *E*) for a first-layer module, the max-cut problem on *G* aims to find a mapping $\textbf{f}: V \mapsto \{0,1\}$ that approximates the binary function of the module.

In general, a conflict graph *G*(*V*, *E*) consists of a set *V* of vertices and a set *E* of pairs of vertices, called edges. We say two vertices $u \in V$ and $v \in V$ are adjacent and conflict with each other if there is an edge $uv \in E$ between them. The basic idea of binary function identification by solving a max-cut problem is to represent the inputs of the binary function as vertices *V* in a graph, and the output of the function as the partition of the vertices into two sets $V^{\prime }$ and $V^{\prime \prime }$. The max-cut problem is used to find the best partition of vertices that maximizes the sum of the weights of the edges connecting the two sets. In other words, for a graph *G*(*V*, *E*) with weights $w_{uv}$ for edge $uv \in E$ between vertices *u* and *v*, the max-cut problem is defined as finding a partition of the vertices *V* into two sets $V^{\prime }$ and $V^{\prime \prime }$ such that the sum of the weights of the edges connecting $V^{\prime }$ and $V^{\prime \prime }$ is maximized. Associating binary variables $x_i$ to every vertex in a graph *G*(*V*, *E*) such that $x_u = 1$ if $u \in V^{\prime }$ and $x_u = 0$ if $u\in V^{\prime \prime }$, the 0-1 quadratic programming formulation of the max-cut problem is given by:

1 $$\begin{aligned} \begin{aligned} \textrm{max} \sum _{\textrm{all} \; (u,v) , \; u<v} w_{uv} (x_u + x_v - 2 x_u x_v), \hspace{1 in} x_u \in \{0, 1\} \;,\; u = 1, \ldots , n , \end{aligned} \end{aligned}$$

where *n* is the number of vertices, and $w_{uv}=0$ if there is no edge between vertices *u* and *v*.

This strategy allows us to identify the input/output (I/O) function of every first-layer module within our hybrid model. We use them to determine the inputs of the output module, which produces a mortality probability. The I/O function of the output module is then identified by a majority voting scheme.

In the next subsection, we introduce our risk stratification algorithm by delving into the process of function identification for the interior black-box modules incorporated within our structured hybrid model. Our aim is to offer a comprehensive description of how both the first-layer and output modules are identified, as this plays a vital role in effectively addressing the classification problem. Furthermore, we outline the algorithm with pseudo-code, providing a clear understanding of its implementation in Python.

### The learning algorithm

**Figure 6:**
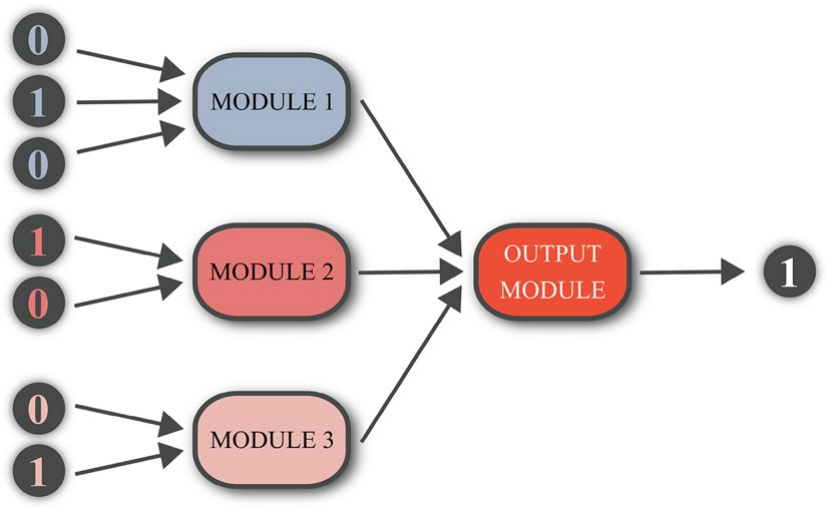
Simple case: a tree-structured network with three first-layer modules mapping 7-dimensional binary input variable to binary outputs.

Here, we present the numerical formulation of the developed mortality risk stratification hybrid model that has been implemented in Python. To make the description more tractable, we focus on a simple case with a series of simplifying assumptions: a two-layered tree-structured network $\textbf{F}_{simple} : \{0, 1\}^7 \longmapsto \{0, 1\}$ with 3 first-layer modules, see Fig. 6.

Given a set of training data $\{(\mathbf {x_s}, y_s) | s = 1,\ldots , S\}$, the risk stratification algorithm receives three parameters: *X*, $\textbf{y}$, and $\textbf{n}$. *X* is a matrix of size $n_{samples} \times n_{features}$ containing all binary-represented input data to $\textbf{F}_{simple}$. In the simple case, $n_{samples} =S$ and $n_{features}=7$, which are the number of data samples and the number of features in the training data, respectively. $\textbf{y}$ is a vector with $n_{samples}$ elements arranged in a single column holding the binary labels of all data samples. Lastly, $\textbf{n}$ is a row vector with 3 elements holding the number of input features to each first-layer module of the network: $\textbf{n} = [3, 2, 2]$.

We define a label function $\textbf{L}$ as follows: assume a tree-structured network $\textbf{F}: \{0, 1\}^N \longmapsto \{0, 1\}$ with *M* first-layer modules. The label function $\textbf{L}: (X, \textbf{y}) \mapsto {T_0}, {T_1}$ receives all binary-represented input data *X* alongside their associated labels $\textbf{y}$ and returns two rank *M* tensors ${T_0}$ and ${T_1}$ each containing $2^N$ elements. Each element of ${T_0}$ and ${T_1}$ embodies a binary input configuration to $\textbf{F}$ and respectively holds the number of 0 and 1 labels for that input configuration in given training data.

**Figure 7:**
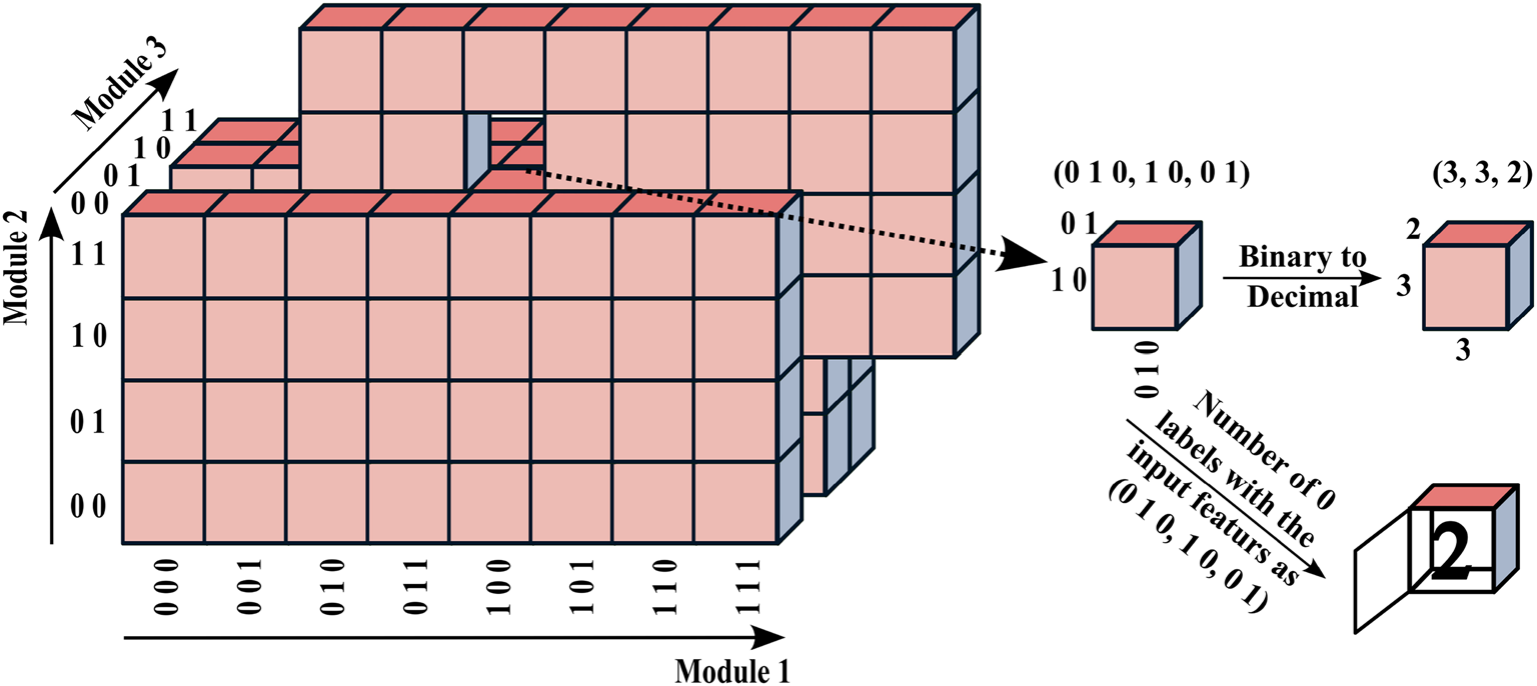
The schematic representation of ${T_0}$ for the simple case, which contains $2^7$ elements holding the number of 0 labels for each input configuration in given training data.

In the simple case, the label function $\textbf{L}_{simple}$ returns two rank 3 tensors, ${T_0}$ and ${T_1}$, since there are three first-layer modules in $\textbf{F}_{simple}$. Each index of ${T_0}$ and ${T_1}$ runs along an axis corresponding to a first-layer module and are constrained by $2^{n_m}$, where $n_m$ is the dimension of the binary input space of the $m\text {th}$ module, and $\sum _{m=1}^3 n_m = 7$. Figure 7 schematically illustrates ${T_0}$ for the simple case. Each element of ${T_0}$ can be accessed by three indexes, e.g., by a set of binary values (0 1 0, 1 0, 0 1) or their corresponding decimal values (3, 3, 2) using the following relation:

2 $$\begin{aligned} Decimal(x_{1} x_{2} \ldots x_{n_m}) = 1 + \sum _{i=1}^{i=n_m} 2^{n_{m}-i} \times x_i. \end{aligned}$$

Algorithm 1 shows the pseudo-code of the risk stratification algorithm for the simple case of Fig. 6, $\textbf{F}_{simple} : \{0, 1\}^7 \longmapsto \{0, 1\}$ with 3 first-layer modules. Lines 3–19 of Algorithm 1 describe nested *for* loops for function identification of the three first-layer modules of $\textbf{F}_{simple}$. The algorithm can be simply generalized for a higher number of first-layer modules by using recursive functions for implementing the *for* loops between lines 3 and 19.

**Algorithm 1:**
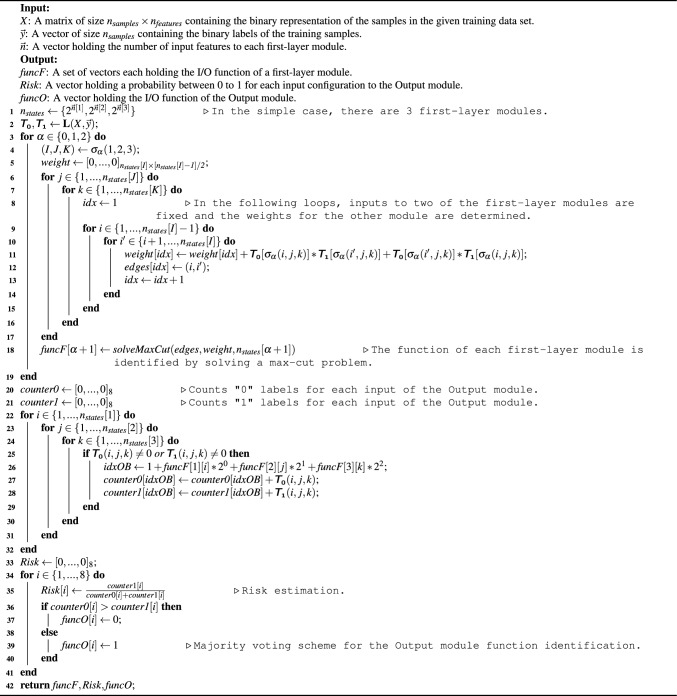
Risk stratification algorithm.

To determine weights of the conflict graphs *G*(*V*, *E*) for the first-layer modules, circular permutations are used to set inputs of two of the first-layer modules fixed and examine the impact of the other first-layer module on the overall output of $\textbf{F}_{simple}$. The circular permutation is a permutation of an ordered set that the elements are shifted by the same amount to the right:

3 $$\begin{aligned} \begin{aligned} \sigma _1 (\{A, B, C\}) = \{C, A, B\} \\ \sigma _2 (\{A, B, C\}) = \{B, C, A\} \end{aligned} \end{aligned}$$

For example, when $(I, J, K) = \sigma _0 (1, 2, 3) = (1, 2, 3)$ in line 4, the nested *for* loops in Lines 6–17 select all pairs of samples in given training data and examine all possible variations of inputs to Module-1 (iterated on *I*) for all fixed inputs to Module-2 and Module-3 (iterated on *J* and *K*, respectively) to specify the weights of the conflict graph $G_1(V_1, E_1)$ for Module-1.

**Figure 8:**
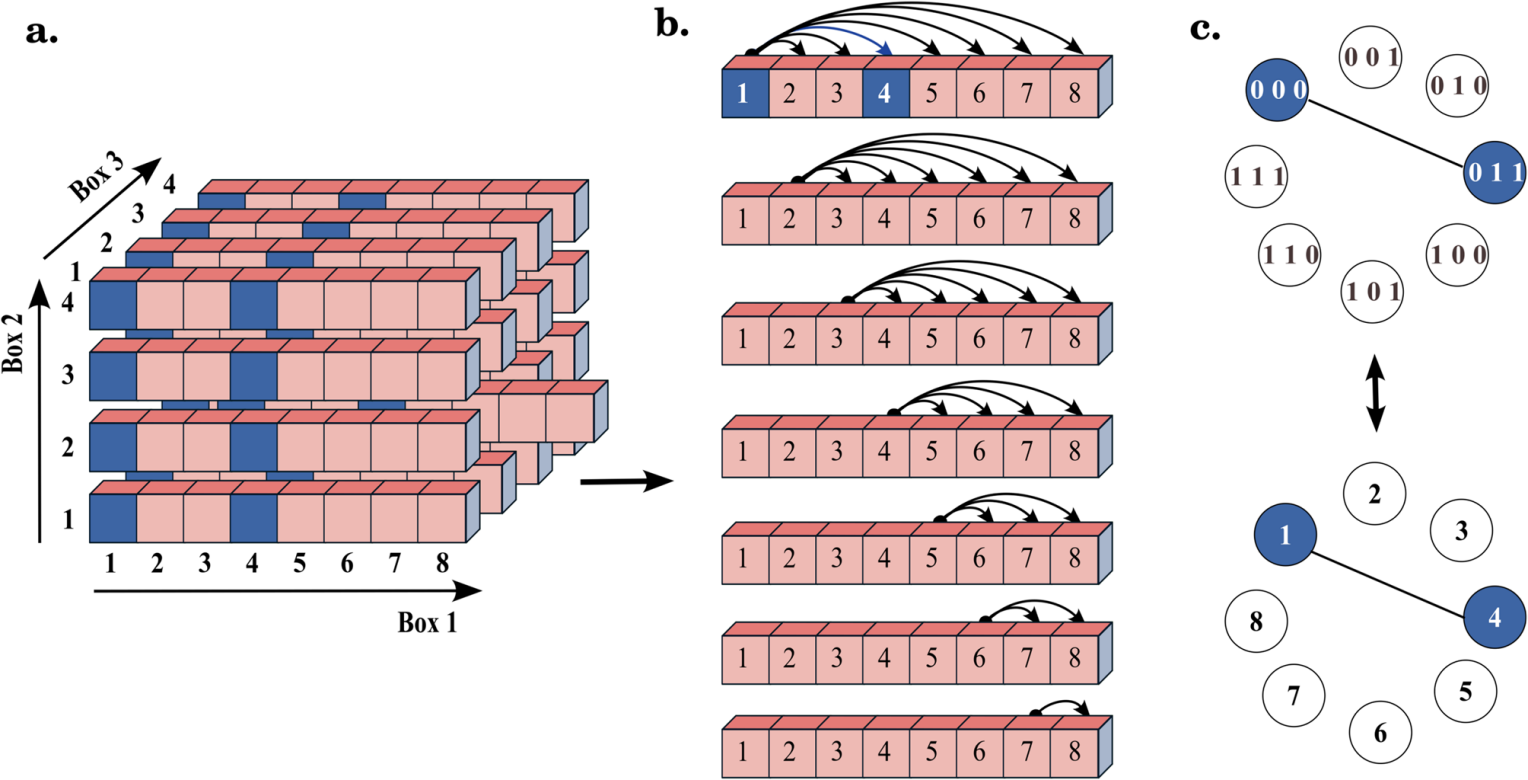
(**a**) All $2^7$ possible binary inputs of $\textbf{F}_{simple}$. Each row runs along 8 input configuration $V_1 = \{1, 2,\ldots , 8\}$ of Module-1 and depicts the inputs variables of $\textbf{F}_{simple}$ with fixed inputs to Module-2 and Module-3. The blue cells in the same row depict all 16 possible pairs of input variables for which the decimal representation of the inputs to the 3 first-layer modules are like (1, *j*, *k*) and (4, *j*, *k*). (**b**) To determine the weights of the conflict graph $G_1(V_1, E_1)$ of Module-1, we compare the labels of input variables within the same row. (**c**) The conflict graph $G_1(V_1, E_1)$ of Module-1 with both binary and decimal representations of vertices. In the risk stratification algorithm, the value of edge $w_{1 4}$ results from Eq. (4) iterated over all $j \in V_2 = \{1, 2, 3, 4\}$ and $k \in V_3 = \{1, 2, 3, 4\}$.

Figure 8 provides a graphical representation of the weight specification for the conflict graph $G_1(V_1, E_1)$ of Module-1 in the case of $\textbf{F}_{simple}$. In Module-1, which consists of 3 input features, we have $2^3$ possible binary input configurations. These configurations are represented in decimal form and form the vertex set $V_1$ of the conflict graph for Module-1, denoted as $V_1 = \{1, 2, \ldots , 8\}$. The elements of $V_1$ serve as the vertices of the conflict graph $G_1(V_1, E_1)$ for Module-1.

To determine the weight between two vertices, such as $w_{1 4} \in E_1$, we consider pairs of samples from the given training data. Specifically, we focus on pairs where the decimal representations of the inputs to the first-layer modules are of the form (1, *j*, *k*) and (4, *j*, *k*), with $j \in V_2 = \{1, 2, 3, 4\}$ and $k \in V_3 = \{1, 2, 3, 4\}$. Next, we calculate the number of such pairs that have different labels. This computation involves the following expression:

4 $$\begin{aligned} {T_0}[1, j, k] \times {T_1}[4, j, k] + {T_0}[4, j, k] \times {T_1}[1, j, k] . \end{aligned}$$

Then, we add the result of Eq. (4) to the value of $w_{1 4}$, see Line 11 of Algorithm 1.

The $solveMaxCut$ function, located in line 18 of Algorithm 1, identifies the I/O function of a first-layer module and stores the result in $funcF[\alpha +1]$, where $\alpha \in \{0, 1, 2\}$ is a counter for the three first-layer modules in $\textbf{F}_{simple}$. The $solveMaxCut$ function achieves this by solving a max-cut problem for the module’s associated conflict graph *G*(*V*, *E*). The primary objective of the $solveMaxCut$ function is to discover a partition of the conflict graph’s vertices into two sets. This partition aims to maximize the number of edges that exist between the two sets. To solve the max-cut problem efficiently, we employed the CVXPY Python package^77,78^. Specifically, we implemented the Goemans–Williamson randomized approximation algorithm by using CVXPY. This algorithm provides a lower bound for the solution of the max-cut problem, estimated at 0.87 times the optimal value.

Now, with access to the I/O function of the first-layer modules, we determine the inputs destined for the output module for each training data sample. The two variables *counter*0 and *counter*1 respectively hold the number of 0 and 1 labels in the given training data for each input of the output module, see lines 20 and 21 in Algorithm 1. The size of *counter*0 and *counter*1 equals $2^3$ as there are three first-layer modules in the simple case. Lines 22–32 in Algorithm 1 perform counting the number of 0 and 1 labels in the training data for each input configuration to the output module.

Proceeding to the last phase, we assign a probability (in this work, the risk of developing a condition—specifically, mortality) to each input of the output module, which subsequently extends to the inputs of $\textbf{F}_{simple}$. This process is captured in lines 33–35 of Algorithm 1, where we define the variable *Risk* that contains the probabilities assigned to each of the $2^3$ inputs directed towards the output module. This is calculated using the following formula:

5 $$\begin{aligned} Risk[i] = \frac{counter1[i]}{counter0[i] + counter1[i]}, \end{aligned}$$

where, the index $i \in \{1,\ldots , 8\}$ refers to a particular input configuration to the output module. Once we’ve established this, we can identify the I/O function of the output module using a majority voting system. By comparing the value of *counter*0 and *counter*1 for each input of the output module, the more probable outcome in the training data is assigned as the output of the output module and stored in the *funcO* variable, see lines 36–40 in Algorithm 1. Finally, the variables *funcF*, *Risk*, and *funcO* represent the outputs of Algorithm 1. These variables determine the I/O functions of the first-layer modules, the mortality risk for the inputs of $\textbf{F}_{simple}$, and the I/O functions of the output module, respectively.

### Mathematical formulation of the learning strategy

This section provides an in-depth exploration of the function identification strategy embedded within each black-box module of the proposed hybrid modeling framework. We begin by introducing the function $\textbf{F}$ of a two-layered tree-structured network with *M* first-layer modules (see Fig. 2 as a practical example):

6 $$\begin{aligned} \textbf{F}: \textbf{x} \in \{0, 1\}^N \longmapsto y \in \{0, 1\}, \end{aligned}$$

where $\textbf{x} \in \{0, 1\}^N$ represents the N-dimensional binary input vector, and $y \in \{0, 1\}$ is the associated output or label. The objective is to deduce the I/O function of all *M* first-layer modules and the output module accurately labeling data points not in the training set, using a given training set of *S* examples $\{(\textbf{x}_s, y_s) | s = 1,\ldots , S\}$.

The set of *N* input features to the tree-structured network in Eq. (6) can be decomposed into *M* vectors, which first-layer modules separately perform computations on:

$$\begin{aligned} \big[ [x_1^1,\ldots , x_1^{n_1}],\ldots , [x_M^1,\ldots , x_M^{n_M}] \big]. \end{aligned}$$

Accordingly, $[x_m^i]_{i=1}^{i=n_m}$ is the subset of input features forwarded to the $m\text {th}$ first-layer module, where $n_m$ is the size of the subset or the dimension of the binary input space of the $m\text {th}$ first-layer module, $\sum _{m=1}^M n_m = N$, and each $x_m^i \in \{0, 1\}$.

The $m\text {th}$ first-layer module involves the conversion of an $n_m$-dimensional binary variable to its associated decimal representation, as illustrated by Eq. (2), before its incorporation. Consequently, an *N*-dimensional binary input vector $\textbf{x} \in \{0, 1\}^N$ can be portrayed as an *M*-dimensional vector $\textbf{X} \in \mathbb {N}^M$:

7 $$\begin{aligned} \textbf{X} = \Big [ Decimal\Big ([x_1^i]_{i=1}^{i=n_1}\Big ), \ldots , Decimal\Big ([x_M^i]_{i=1}^{i=n_M} \Big ) \Big ]. \end{aligned}$$

The decimal subset $V_m = \{v_m^k\}_{k=1}^{k=2^{n_m}}$ of the $m\text {th}$ first-layer module is defined as a set that holds the decimal representations of all $2^{n_m}$ binary configurations of $[x_m^i]_{i=1}^{i=n_m}$. In other words, $V_m = \{v_m^1, v_m^2, v_m^3, \ldots , v_m^{2^{n_m}}\} = \{1, 2, 3, \ldots , 2^{n_m}\}$. Therefore, the function $\textrm{F}_m$ of the $m\text {th}$ first-layer module, receives a decimal value in $V_m$ and forwards a binary value $f_m \in \{0, 1\}$ to the output module:

8 $$\begin{aligned} f_m = {\textrm{F}}_m(Decimal\Big ([x_m^i]_{i=1}^{i=n_m}\Big )), \qquad x_m^i \;, f_m \in \{0, 1\}, \qquad {\textrm{F}}_m : V_m \longmapsto f_m, \end{aligned}$$

where $m \in \{1, 2,\ldots , M\}$. Then, the output module receives an M-dimensional binary variable from all *M* first-layer modules. After converting it to a decimal value, which is in the decimal subset $V_O = \{1, 2, 3,\ldots , 2^{M}\}$ of the output module, the function of the output module $\textrm{F}_O$ returns the predicted label:

9 $$\begin{aligned} y = {\textrm{F}}_O(Decimal\Big ([f_i]_{i=1}^{i=M}\Big )), \qquad f_i \;, y \in \{0, 1\}, \qquad {\textrm{F}}_O : V_O \longmapsto y. \end{aligned}$$

We define a characteristic graph *G*(*V*, *E*) for each first-layer module of the tree-structured network under consideration. This allows us to employ graph-theoretic methods to deduce its I/O function. Specifically, we utilize a provided set of training data and map the identification of individual interior black-box modules to the solving of max-cut problems.

The characteristic graph $G_m(V_m, E_m)$ for the $m\text {th}$ first-layer module is defined by considering $V_m$ as the decimal subset of the $m\text {th}$ first-layer module. In this graph, an edge $v_m^k v_m^l$ belongs to $E_m$ with a weight of $w_{m}^{kl}$ to represent the dissimilarity between the function $F_m$ of the $m\text {th}$ first-layer module for the associated vertices $v_m^k$ and $v_m^l$.

Hereafter, we explore how to discern disparities in the output s of a first-layer module for two distinct vertices in the corresponding characteristic graph, e.i., ${\textrm{F}}_m(v_m^k) \ne {\textrm{F}}_m(v_m^l)$. Furthermore, we explore the process of determining edge weights $w_{m}^{kl}$ within a characteristic graph. This step lays the groundwork for leveraging solutions to a max-cut problem to identify the function associated with the first-layer module.

Consider two input samples $\textbf{p}, \textbf{q} \in \{0, 1\}^N$ to the Eq. (6), for which the inputs to all first-layer modules except for the $m\text {th}$ first-layer module are identical:

10 $$\begin{aligned} \exists ! \; m \in \{1, \ldots , M\} \; \ni \; [p_m^i]_{i=1}^{i=n_m} \ne [q_m^i]_{i=1}^{i=n_m}. \end{aligned}$$

For $v_m^k = Decimal\Big ([p_m^i]_{i=1}^{i=n_m}\Big )$ and $v_m^l = Decimal\Big ([q_m^i]_{i=1}^{i=n_m}\Big )$, $\textrm{F}_m(v_m^k) \ne \textrm{F}_m(v_m^l)$ if and only if $\textbf{p}$ and $\textbf{q}$ have different labels $y_{\textbf{p}} \ne y_{\textbf{q}}$. In simpler terms, according to Eq. (8), $\textrm{F}_m(v_m^k)$ and $\textrm{F}_m(v_m^l)$ yield either 0 or 1. These, along with the outputs of the remaining $M-1$ first-layer modules, are then directed to the output module for the final computation towards the labels. As per the mathematical definition, a function produces a unique output for a given input. Therefore, if two input samples $\textbf{p}$ and $\textbf{q}$ share identical inputs for all first-layer modules except the $m\text {th}$ one, the corresponding outputs of the first-layer modules must also be identical. Any disparity in the output module results, i.e., $y_{\textbf{p}} \ne y_{\textbf{q}}$, implies differing inputs to the output module. This dissimilarity can be attributed solely to the output of the $m\text {th}$ first-layer module: $\textrm{F}_m(v_m^k) \ne \textrm{F}_m(v_m^l)$, as the outputs of the remaining $M-1$ first-layer modules must be consistent.

Finally, we provide a concise explanation of the process for assigning weights to the edges of characteristic graphs. In a graph *G*(*V*, *E*), edges *E* can be endowed with weights *W* to indicate the significance or strength of the connection between the two vertices linked by the edge.

As elucidated earlier, an edge in a characteristic graph signifies that the outputs of the first-layer module differ for the respective vertices serving as inputs. We employed a pair of input samples to establish an edge between two vertices in a characteristic graph. However, this process can be extended to encompass all $$\left( {\begin{array}{c}S\\ 2\end{array}}\right)$$ pairs of input samples $\textbf{p}$ and $\textbf{q}$ within a given training dataset. If the chosen pairs exhibit distinct labels and satisfy the condition (10), the weight of the corresponding edge in $G_m(V_m, E_m)$ is incremented by one. In other words, we define a $2^{n_m} \times 2^{n_m}$ weight matrix $W_m$ for the $m\text {th}$ first-layer module by:

11 $$\begin{aligned} W_m = 0_{2^{n_m}, 2^{n_m}} + \sum _{\mathrm {all\;pairs\;(\textbf{p},\textbf{q})}} |y_{\textbf{p}} - y_{\textbf{q}}| \times \prod _{i=1\;,\;i \ne m}^{i=M} \delta (\textbf{P}[i] - \textbf{Q}[i]) \times \textbf{e}_{\textbf{P}[i]} \textbf{e}_{\textbf{Q}[i]}^T , \end{aligned}$$

where $\textbf{P}, \textbf{Q} \in \mathbb {N}^M$ are M-dimensional decimal representations of binary input vectors $\textbf{p}, \textbf{q} \in \{0, 1\}^N$ with labels $y_{\textbf{p}}, y_{\textbf{q}} \in \{0, 1\}$, $\delta$ is the Kronecker delta function, and $\textbf{e}_i$ are elements of the standard basis of vector space $\mathbb {R}^{2^{n_m}}$:

12 $$\begin{aligned} \textbf{e}_1 = [1, 0, 0, \ldots , 0]^T \;,\; \textbf{e}_2 = [0, 1, 0, \ldots , 0]^T \;,\; \ldots \;,\; \textbf{e}_{2^{n_m}} = [0, 0, 0, \ldots , 1]^T . \end{aligned}$$

Once the weight matrices of all *M* characteristic graphs have been determined using the available training data, we proceed to identify the functions associated with the first-layer modules by partitioning the vertices of the characteristic graphs into two sets. The solution to the max-cut problem illustrated in Eq. (1) is employed to determine the optimal partition of vertices, maximizing the sum of weights for edges connecting the two sets. The function of the output module is then identified by a majority voting scheme as shown in Eq. (5).

### Ethical approval

All experimental protocols were approved by the Ethics Committee of the RWTH Aachen Faculty of Medicine (local Ethics Committee reference number: EK 102/19, date of approval: 26.03.2019). As well, the Ethics Committee of the RWTH Aachen Faculty of Medicine (local Ethics Committee reference number: EK 102/19, date of approval: 26.03.2019) waived the need to obtain Informed consent for the collection and retrospective analysis of the de-identified data as well as the publication of the results of the analysis. All methods were performed in accordance with the relevant guidelines and regulations.

### Supplementary Information

Supplementary Information.

## Data Availability

The data included in this study, contain sensitive health-related information. Due to the small data set, anonymisation techniques, like e.g. k-anonymity, cannot be applied usefully without a relevant loss of information. Thus, according to the Health Data Protection Act North Rhine-Westphalia (Gesundheitsdatenschutzgesetz NRW) and the internal guidelines of the Data Protection Officer of the University Hospital RWTH Aachen, the raw patient data must not be made publicly available, since a total anonymisation cannot be guaranteed. However, researchers who are interested in the data, may send their informal request to the Department of Intensive Care Medicine (Email: oim@ukaachen.de) of the University Hospital RWTH Aachen with a statement which research questions they aim at and which data are necessary for this purpose. Then, in a bilateral process, a solution for the data exchange can be found in compliance with legal and ethical restrictions.

## References

[1] Sekulic AD, Trpkovic SV, Pavlovic AP, Marinkovic OM, Ilic AN (2015). Scoring systems in assessing survival of critically ill ICU patients. Med. Sci. Monit. Int. Med. J. Exp. Clin. Res..

[2] Kafan S (2021). Predicting risk score for mechanical ventilation in hospitalized adult patients suffering from covid-19. Anesthesiol. Pain Med..

[3] Verburg IWM (2017). Which models can i use to predict adult ICU length of stay? A systematic review. Crit. Care Med..

[4] Rapsang AG, Shyam DC (2014). Scoring systems in the intensive care unit: A compendium. Indian J. Crit. Care Med. Peer Rev..

[5] Knaus WA, Zimmerman JE, Wagner DP, Draper EA, Lawrence DE (1981). Apache-acute physiology and chronic health evaluation: A physiologically based classification system. Crit. Care Med..

[6] Le Gall J-R (1984). A simplified acute physiology score for ICU patients. Crit. Care Med..

[7] Vincent, J. L. *et al.* The sofa (sepsis-related organ failure assessment) score to describe organ dysfunction/failure: On behalf of the working group on sepsis-related problems of the European society of intensive care medicine (see contributors to the project in the appendix) (1996). 10.1007/BF01709751 8844239

[8] Ferreira FL, Bota DP, Bross A, Mélot C, Vincent J-L (2001). Serial evaluation of the sofa score to predict outcome in critically ill patients. JAMA.

[9] Huang X (2022). Risk assessment of ICU patients through deep learning technique: A big data approach. J. Glob. Health.

[10] Wasilewski P (2020). Covid-19 severity scoring systems in radiological imaging—a review. Pol. J. Radiol..

[11] Barnett, A. J. *et al.* Iaia-bl: A case-based interpretable deep learning model for classification of mass lesions in digital mammography. arXiv:2103.12308 (arXiv preprint) (2021).

[12] Garcia PDW (2020). Prognostic factors associated with mortality risk and disease progression in 639 critically ill patients with covid-19 in Europe: Initial report of the international RISC-19-ICU prospective observational cohort. EClinicalMedicine.

[13] Ryan L (2020). Mortality prediction model for the triage of covid-19, pneumonia, and mechanically ventilated ICU patients: A retrospective study. Ann. Med. Surg..

[14] O’malley KJ (2005). Icd code accuracy. Measuring diagnoses. Health Serv. Res..

[15] Schinkel M, Paranjape K, Panday RN, Skyttberg N, Nanayakkara PW (2019). Clinical applications of artificial intelligence in sepsis: A narrative review. Comput. Biol. Med..

[16] Alcaide D, Aerts J (2021). A visual analytic approach for the identification of ICU patient subpopulations using ICD diagnostic codes. PeerJ Comput. Sci..

[17] Harerimana G, Kim JW, Jang B (2021). A deep attention model to forecast the length of stay and the in-hospital mortality right on admission from ICD codes and demographic data. J. Biomed. Inform..

[18] Chen L (2009). Curse of Dimensionality.

[19] Altman N, Krzywinski M (2018). The curse (s) of dimensionality. Nat. Methods.

[20] Bartley ML, Hanks EM, Schliep EM, Soranno PA, Wagner T (2019). Identifying and characterizing extrapolation in multivariate response data. PLoS ONE.

[21] Barbiero, P., Squillero, G. & Tonda, A. Modeling generalization in machine learning: A methodological and computational study. arXiv:2006.15680 (arXiv preprint) (2020).

[22] Rudin C (2022). Interpretable machine learning: Fundamental principles and 10 grand challenges. Stat. Surv..

[23] Barnett AJ (2021). A case-based interpretable deep learning model for classification of mass lesions in digital mammography. Nat. Mach. Intell..

[24] Chen C (2019). This looks like that: Deep learning for interpretable image recognition. Adv. Neural. Inf. Process. Syst..

[25] Fröhlich H (2018). From hype to reality: Data science enabling personalized medicine. BMC Med..

[26] Singer M (2016). The third international consensus definitions for sepsis and septic shock (sepsis-3). JAMA.

[27] Li J (2022). Predicting mortality in intensive care unit patients with heart failure using an interpretable machine learning model: Retrospective cohort study. J. Med. Internet Res..

[28] Qiu W (2022). Interpretable machine learning prediction of all-cause mortality. Commun. Med..

[29] Quanjel MJ (2021). Replication of a mortality prediction model in Dutch patients with covid-19. Nat. Mach. Intell..

[30] Barish M, Bolourani S, Lau LF, Shah S, Zanos TP (2021). External validation demonstrates limited clinical utility of the interpretable mortality prediction model for patients with covid-19. Nat. Mach. Intell..

[31] Singh H, Mhasawade V, Chunara R (2022). Generalizability challenges of mortality risk prediction models: A retrospective analysis on a multi-center database. PLoS Digital Health.

[32] Sharafutdinov K (2023). Computational simulation of virtual patients reduces dataset bias and improves machine learning-based detection of ARDS from noisy heterogeneous ICU datasets. IEEE Open J. Eng. Med. Biol..

[33] Sharafutdinov K (2022). Application of convex hull analysis for the evaluation of data heterogeneity between patient populations of different origin and implications of hospital bias in downstream machine-learning-based data processing: A comparison of 4 critical-care patient datasets. Front. Big Data.

[34] Chu J (2021). Knowledge-aware multi-center clinical dataset adaptation: Problem, method, and application. J. Biomed. Inform..

[35] Collins GS, Reitsma JB, Altman DG, Moons KG (2015). Transparent reporting of a multivariable prediction model for individual prognosis or diagnosis (tripod): The tripod statement. Ann. Intern. Med..

[36] Wolff RF (2019). Probast: A tool to assess the risk of bias and applicability of prediction model studies. Ann. Intern. Med..

[37] Von Stosch M, Oliveira R, Peres J, de Azevedo SF (2014). Hybrid semi-parametric modeling in process systems engineering: Past, present and future. Comput. Chem. Eng..

[38] Samadi E, Kiefer M, Fritsch S, Bickenbach SJ, Schuppert A (2022). A training strategy for hybrid models to break the curse of dimensionality. PLoS One.

[39] Schuppert AA (2000). Extrapolability of structured hybrid models: A key to optimization of complex processes. Equadiff 99: (In 2 Volumes).

[40] Fiedler B, Schuppert A (2008). Local identification of scalar hybrid models with tree structure. IMA J. Appl. Math..

[41] Glassey J, Von Stosch M (2018). Hybrid Modeling in Process Industries.

[42] Procopio A (2023). Combined mechanistic modeling and machine-learning approaches in systems biology—a systematic literature review. Comput. Methods Programs Biomed..

[43] Marx G (2021). Algorithmic surveillance of ICU patients with acute respiratory distress syndrome (ASIC): Protocol for a multicentre stepped-wedge cluster randomised quality improvement strategy. BMJ Open.

[44] Winter A (2018). journalSmart medical information technology for healthcare (smith). Methods Inf. Med..

[45] Hirsch J (2016). Icd-10: History and context. Am. J. Neuroradiol..

[46] Gupta M (2022). An extensive data processing pipeline for mimic-iv. Machine Learning for Health.

[47] Debray TP (2015). A new framework to enhance the interpretation of external validation studies of clinical prediction models. J. Clin. Epidemiol..

[48] Virtanen P (2020). SciPy 1.0. Fundamental algorithms for scientific computing in Python. Nat. Methods.

[49] Seabold, S. & Perktold, J. statsmodels: Econometric and statistical modeling with python. In *9th Python in Science Conference* (2010).

[50] Poston JT, Koyner JL (2019). Sepsis associated acute kidney injury. BMJ.

[51] Peerapornratana S, Manrique-Caballero CL, Gómez H, Kellum JA (2019). Acute kidney injury from sepsis: Current concepts, epidemiology, pathophysiology, prevention and treatment. Kidney Int..

[52] Zarbock A (2023). Sepsis-associated acute kidney injury: Consensus report of the 28th acute disease quality initiative workgroup. Nat. Rev. Nephrol..

[53] Bajpai VK (2019). Invasive fungal infections and their epidemiology: Measures in the clinical scenario. Biotechnol. Bioprocess Eng..

[54] Saied WI (2019). A comparison of the mortality risk associated with ventilator-acquired bacterial pneumonia and nonventilator ICU-acquired bacterial pneumonia. Crit. Care Med..

[55] Nasir N (2023). Comparison of risk factors and outcome of patients with and without covid-19-associated pulmonary aspergillosis from Pakistan: A case–control study. Mycoses.

[56] Huang Y-F (2020). A population-based cohort study of mortality of intensive care unit patients with liver cirrhosis. BMC Gastroenterol..

[57] Kartoun U (2017). The meld-plus: A generalizable prediction risk score in cirrhosis. PLoS One.

[58] Bajaj JS, O’Leary JG, Wong F, Reddy KR, Kamath PS (2012). Bacterial infections in end-stage liver disease: Current challenges and future directions. Gut.

[59] Verma N (2022). Factors determining the mortality in cirrhosis patients with invasive candidiasis: A systematic review and meta-analysis. Med. Mycol..

[60] Rosenthal VD (2022). The impact of healthcare-associated infections on mortality in ICU: A prospective study in Asia, Africa, Eastern Europe, Latin America, and the middle east. Am. J. Infect. Control.

[61] Gupta M, Maiwall R (2023). Acute on chronic liver failure: An update. Peri-operative Anesthetic Management in Liver Transplantation.

[62] Essing T (2023). Clinical determinants of hospital mortality in liver failure: A comprehensive analysis of 62,717 patients. Z. Gastroenterol..

[63] Diez-Quevedo C (2021). Mental disorders, psychopharmacological treatments, and mortality in 2150 covid-19 Spanish inpatients. Acta Psychiatr. Scand..

[64] Liu NH (2017). Excess mortality in persons with severe mental disorders: A multilevel intervention framework and priorities for clinical practice, policy and research agendas. World Psychiatry.

[65] Vai B (2021). Mental disorders and risk of covid-19-related mortality, hospitalisation, and intensive care unit admission: A systematic review and meta-analysis. Lancet Psychiatry.

[66] Oud L, Garza J (2022). Impact of history of mental disorders on short-term mortality among hospitalized patients with sepsis: A population-based cohort study. PLoS One.

[67] Kotfis K, Marra A, Ely EW (2018). ICU delirium—a diagnostic and therapeutic challenge in the intensive care unit. Anaesthesiol. Intensive Ther..

[68] Huapaya JA, Wilfong EM, Harden CT, Brower RG, Danoff SK (2018). Risk factors for mortality and mortality rates in interstitial lung disease patients in the intensive care unit. Eur. Respir. Rev..

[69] Fuchs L (2019). The effect of ARDS on survival: Do patients die from ARDS or with ARDS?. J. Intensive Care Med..

[70] DiSilvio B (2019). Complications and outcomes of acute respiratory distress syndrome. Crit. Care Nurs. Q..

[71] Tuan W-J, Lennon RP, Zhang A, Macherla A, Zgierska AE (2022). Risks of severe covid-19 outcomes among patients with diabetic polyneuropathy in the united states. J. Public Health Manage. Pract..

[72] Amaya-Villar R, Garnacho-Montero J, Ortìz-Leyba C, Márquez-Vácaro JA (2006). Polyneuropathy and discontinuation from mechanical ventilation. Clin. Pulmonary Med..

[73] Chen, T. & Guestrin, C. Xgboost: A scalable tree boosting system. In *Proceedings of the 22nd ACM SIGKDD International Conference on Knowledge Discovery and Data Mining*, 785–794 (2016).

[74] Rudin C (2019). Stop explaining black box machine learning models for high stakes decisions and use interpretable models instead. Nat. Mach. Intell..

[75] Lundberg SM, Lee S-I (2017). A unified approach to interpreting model predictions. Adv. Neural Inf. Process. Syst..

[76] Rodríguez-Fdez, I., Canosa, A., Mucientes, M. & Bugarín, A. STAC: A web platform for the comparison of algorithms using statistical tests. In *Proceedings of the 2015 IEEE International Conference on Fuzzy Systems (FUZZ-IEEE)* (2015).

[77] Agrawal A, Verschueren R, Diamond S, Boyd S (2018). A rewriting system for convex optimization problems. J. Control Decis..

[78] Diamond S, Boyd S (2016). CVXPY: A Python-embedded modeling language for convex optimization. J. Mach. Learn. Res..

