# Supplementary material for: A hybrid modeling framework for generalizable and interpretable predictions of ICU mortality across multiple hospitals
Source: Sci Rep. 2024 Mar 8;14:5725. doi: 10.1038/s41598-024-55577-6 (PMC10923850; doi:10.1038/s41598-024-55577-6)
Supplement: Supplementary file 1 — Supplementary Information. [file 41598_2024_55577_MOESM1_ESM.pdf]

Supplementary Information

A Hybrid Modeling Framework for Generalizable and Interpretable Predictions of ICU Mortality in Mechanically Ventilated Influenza Patients and Pneumonia across Multiple Hospitals Using ICD Codes
Samadi et al. (2024)

1 Jaccard similarity

The generalizability of a clinical prediction model is commonly tested by how well it works with new patient data, evaluated through an external validation study. However, the term "external validation" is loosely defined and can leave ambiguity regarding how different the derivation sample is from the validation sample[1]. To quantify to what extent the derivation and validation samples are related, we used the Jaccard similarity that measures the case mix between the derivation and validation samples.

Jaccard similarity provides a simple, yet effective approach that determines the degree of relatedness between two sets of data. Given two sets A (derivation sample) and B (validation sample), the Jaccard similarity index J is calculated as:

$$J(A,B) = \frac{|A \cap B|}{|A \cup B|} \tag{1}$$

Where:

- $|A \cap B|$ is the count of shared attributes between the derivation and validation samples (intersection),
- $|A \cup B|$ is the count of all attributes present in either the derivation or validation samples, or both (union).

The value of $J(A,B)$ will range from 0 to 1, with 0 indicating no overlap or similarity, and 1 indicating complete overlap or similarity between the samples.

2 Mortality prediction modeling with SOFA scores

To contrast our developed hybrid modeling framework for predicting ICU patient mortality with existing ICU mortality prediction models, we employed the Sequential Organ Failure Assessment (SOFA) score[2], excluding the Glasgow Coma Scale (GCS) score and urine output due to data limitations. The selected physiological parameters used for calculating the SOFA score, obtained within 24 hours of ICU admission, are detailed in Supplementary Table S1.

Supplementary Table S1. Physiological parameters required for calculating the SOFA score (obtained within 24h of ICU admission). Values are represented as mean (standard deviation).

	**Derivation Hospital**	**Validation Hospital 1**	**Validation Hospital 2**	**Validation Hospital 3**	**Validation Hospital 4**
PaO_2/FiO_2 (mmHg)	255.6 (108.0)	304.3 (132.3)	239.9 (110.7)	255.1 (85.7)	270.2 (101.1)
Platelets ($\times 10^3/\mu l$)	215.2 (115.1)	210.4 (113.6)	229.1 (123.7)	210.4 (109.8)	204.8 (109.1)
Bilirubin (mg/dl)	19.6 (42.4)	18.9 (28.3)	19.9 (32.1)	16.4 (20.2)	16.2 (30.6)
Mean arterial pressure (mmHg)	76.5 (9.6)	76.7 (9.5)	76.6 (14.3)	78.0 (8.8)	75.0 (11.2)
Creatinine (μmol/L)	121.8 (102.1)	127.6 (92.2)	137.0 (105.1)	104.5 (77.1)	110.1 (88.3)
Dobutamine (μg/kg/min)	0.0 (0.0)	0.4 (1.5)	0.0 (0.0)	0.7 (1.5)	0.1 (0.7)
Epinephrine (μg/kg/min)	0.0 (0.0)	0.0 (0.0)	0.5 (0.6)	0.0 (0.0)	0.0 (0.8)
Norepinephrine (μg/kg/min)	0.0 (0.0)	0.1 (0.3)	2.7 (7.9)	0.1 (0.3)	0.1 (0.9)
SOFA score (without GCS Score & Urine output variable)	6.5 (2.1)	9.6 (2.2)	7.2 (2.4)	8.6 (2.5)	7.7 (2.6)

To assess the effectiveness of a mortality prediction model utilizing SOFA scores, we employed logistic regression. This statistical methodology leverages the inherent association between SOFA scores and the likelihood of ICU mortality.

Supplementary Figure S1 depicts receiver operating characteristic (ROC) curves illustrating the discriminative performance of the logistic regression model across multiple hospitals. Each curve corresponds to a different hospital, with the Area Under the Curve (AUC) serving as a metric for the model's discriminatory ability. Despite the rationale behind employing the SOFA score, the AUC values reveal suboptimal discriminative performance across all hospitals.

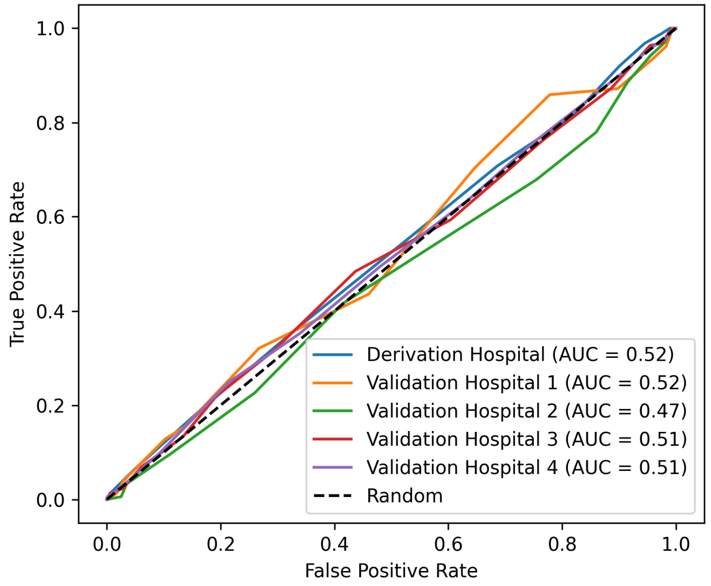

Supplementary Figure S1. ROC curves illustrating the discriminative performance of a logistic regression model utilizing SOFA scores to predict patient ICU mortality across multiple hospitals. Each curve represents a different hospital, with the AUC quantifying the model's discriminatory ability. The proximity of the AUCs to that of a random classifier suggests poor discriminative performance across all hospitals.

3 Quantitative bias analysis

In this section, we present a detailed visualization of the quantitative bias analysis conducted on the clinical datasets outlined in the Table 1 of the main manuscript. This analysis aimed to assess potential biases between Derivationn and Validation Hospitals across various clinical features.

Supplementary Figure S2 illustrates the distribution of continuous variables, including days of ICU stay, age, and BMI, providing a visualized comparison in the distributions between Derivationn and Validation Hospitals. Mann-Whitney U tests were applied to assess disparities in these binary features represented in the Table 1 of the main manuscript. It is noteworthy that the age values has been anonymized to protect the privacy of the study's patients.

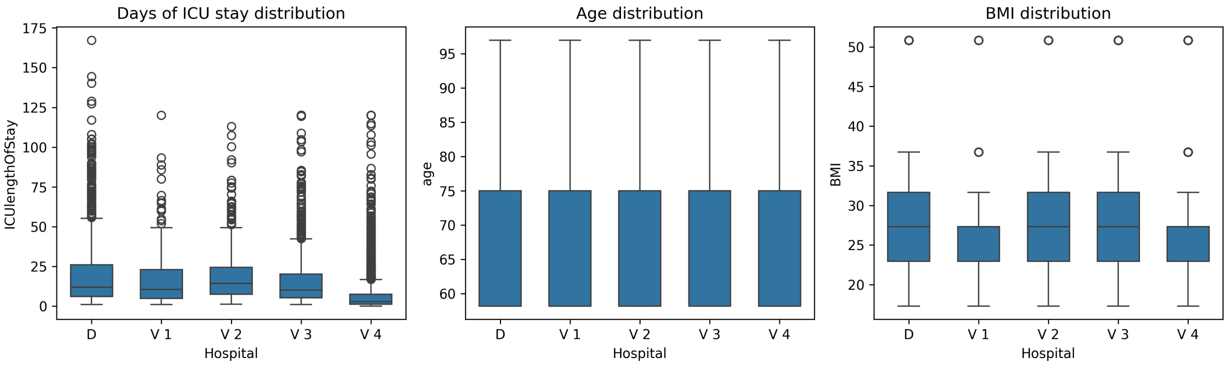

Supplementary Figure S2. Visualized comparison in the distributions for continuous variables, including days of ICU stay, age, and BMI. Mann-Whitney U tests were employed in the Table 1 of the main manuscript to assess distribution disparities between Derivationn and Validation Hospitals. Age values has been anonymized for privacy protecting.

Supplementary Figure S3 expands on the bias analysis for characteristics and demographics features such as mortality, female gender, diabetes mellitus, and thoracic trauma. Proportions z-tests were applied to assess disparities in these binary features in the Table 1 of the main manuscript. The visualization enhances the interpretation of potential biases in these critical clinical attributes.

Additionally, Supplementary Figure S4 provides a visual analysis of bias related to the ICD codes used as binary features in our hybrid modeling framework. This figure facilitates an understanding of distribution disparities among various hospital groups. Furthermore, proportions z-tests were applied to assess disparities in these binary features, as detailed in Table 1 of the main manuscript.

Specifically, the mortality rates depicted in Supplementary Figure S3 across all hospitals appear notably high, underscoring the severity of conditions within the study cohorts. Given that the study focuses on mechanically ventilated influenza patients in the ICU, these rates reflect the critical condition of the cases. Derivationn Hospital shows a mortality rate of 31.6%, with similar rates observed in Derivation Hospitals 1, 3, and 4 ranging from 23.1% to 30.7%. Notably, Validation Hospital 2 exhibits the highest mortality rate at 64.2% (358 out of 558 patients). Supplementary Figure S4 further emphasizes the severity of patient conditions, particularly evident in Validation Hospital 3, where critical conditions such as renal failure, sepsis, liver failure, and ARDS exhibit notably high prevalence.

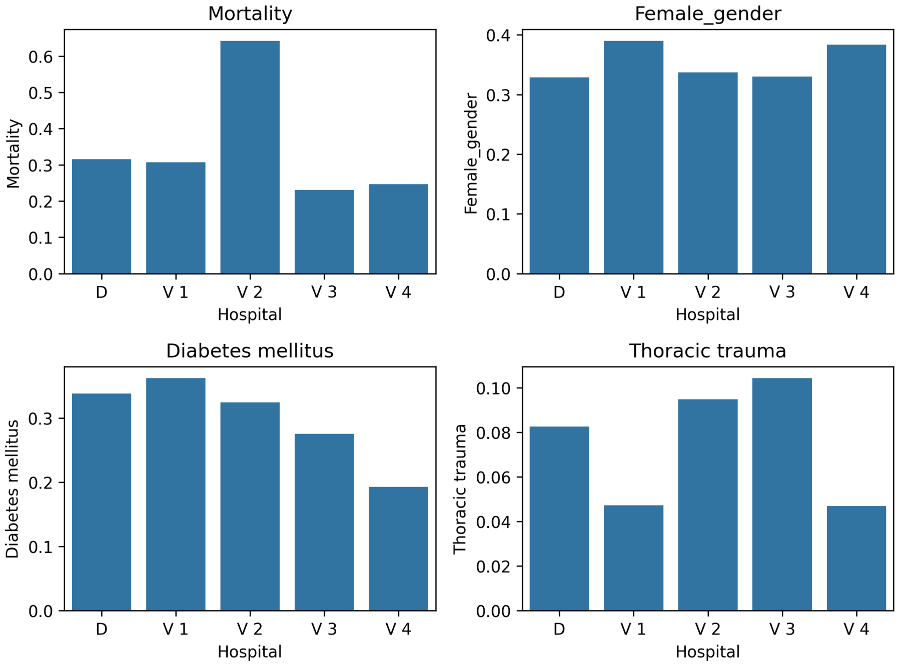

Supplementary Figure S3. Visual representation illustrating the bias analysis for characteristics and demographics features mortality, female gender, diabetes mellitus, and thoracic trauma. Proportions z-tests were utilized in Table 1 of the main manuscript to evaluate disparities between Derivationn and Validation Hospitals.

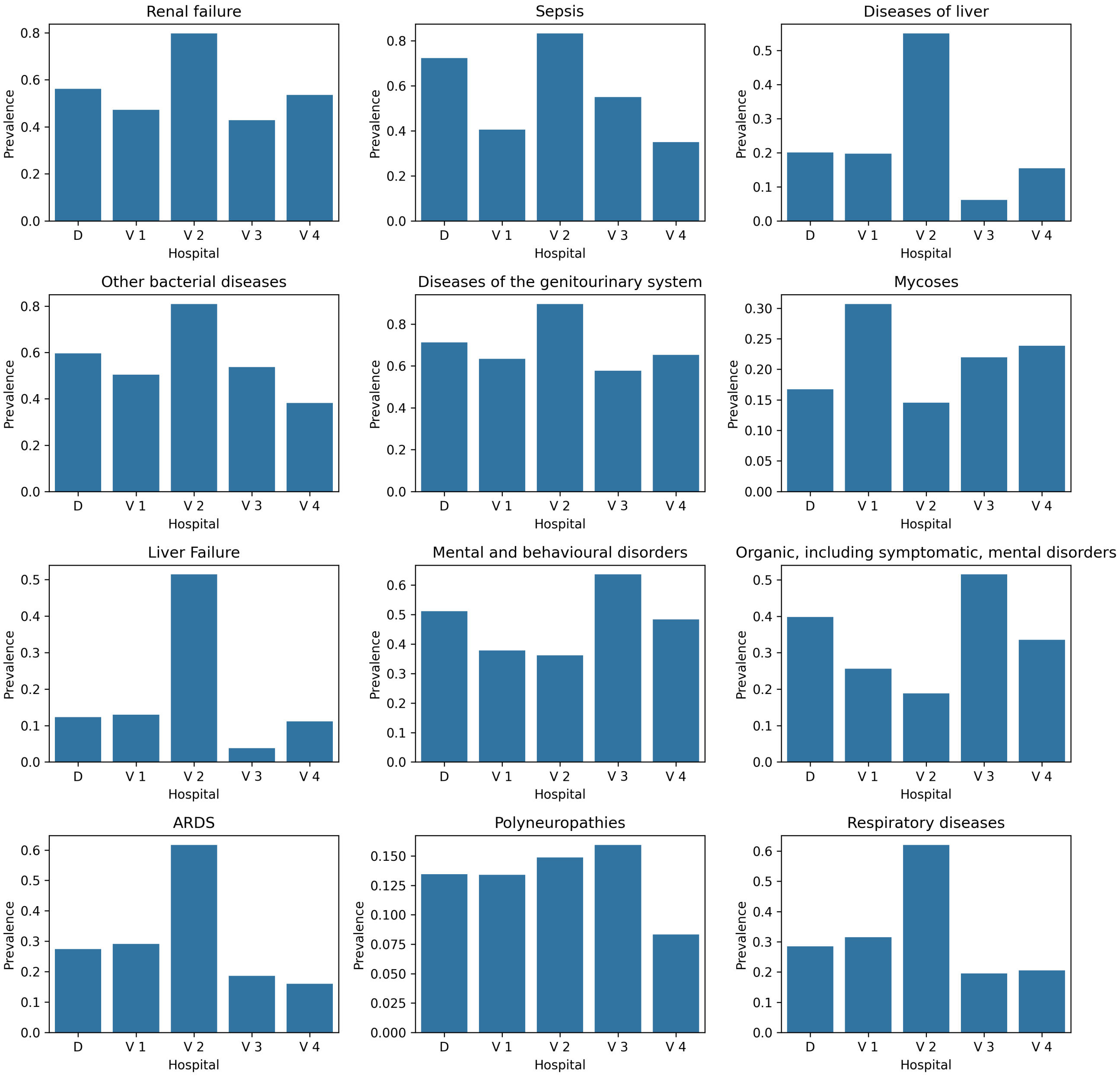

Supplementary Figure S4. Visual representation illustrating the bias analysis for ICD codes used as features in the hybrid modeling framework such as renal failure, sepsis and ARDS. Proportions z-tests were utilized in Table 1 of the main manuscript to evaluate disparities between Derivation and Validation Hospitals.

4 Heart failure module

The deliberate exclusion of specific potential causes of mortality, such as heart failure, from the tree-structured network depicted in Figure 2 in the main manuscript aimed to enhance the precision and relevance of our study on mortality prediction among critically ill ICU patients particularly within the five German hospitals involved in this study. Despite the acknowledged significance of heart failure in the broader medical context, the incorporation of a heart failure module into our first-layer modules revealed limited discriminative value specific to our dataset.

Supplementary Table S2 provides an overview of heart failure-related features, encompassing aspects from patient medical history and those reflected in the ICD codes of the studied patient cohorts across the five German hospitals involved in this study.

Supplementary Table S2. Heart failure related features of the studied patient cohorts from five German hospitals. Variable distributions are reported as n (%).

Heart Failure Related Features	Derivation Hospital	Validation Hospital 1	Validation Hospital 2	Validation Hospital 3	Validation Hospital 4
Chronic Heart Failure	781 (56.1%)	97 (6.9%)	200 (14.3%)	456 (32.7%)	993 (71.3%)
Ischaemic heart diseases (L20-L25)	507 (36.4%)	53 (3.8%)	87 (6.2%)	335 (24.0%)	690 (49.6%)
Pulmonary heart disease and diseases of pulmonary circulation (L26-L28)	259 (18.6%)	8 (0.5%)	90 (6.4%)	117 (8.4%)	130 (9.3%)
Other forms of heart disease (L30-L52)	950 (68.2%)	131 (9.4%)	343 (24.6%)	607 (43.6%)	1156 (83.1%)

In our exploration of the discriminability of binary heart failure-related features highlighted in Supplementary Table S2, a crucial metric employed is the Point-Biserial Correlation Coefficient r_{pb}. This metric quantifies the strength and direction of the association between a binary feature and a target variable, in our case, mortality of the ICU patients.

Supplementary Table S3 provides the representation of r_{pb} for binary heart failure-related features and their correlation with patient mortality, categorized by hospital. Interpretation of this coefficient involves recognizing that a value close to 0 suggests a weak association, while values close to 1 or -1 indicate a strong association. Additionally, the *P* value accompanying the coefficient denotes the statistical significance of the association; a low correlation coefficient with a high *P* value suggests a weak and possibly non-discriminative association.

Supplementary Table S3. The representation of r_{pb} for binary heart failure-related features and their correlation with patient mortality, categorized by hospital. A value of r_{pb} close to 0 suggests a weak association, while values close to 1 or -1 indicate a strong association. *P* values < 0.05 indicate significant difference in the mean mortality for the two groups defined by the binary heart failure-related feature.

Heart Failure Related Features	Significance	Derivation Hospital	Validation Hospital 1	Validation Hospital 2	Validation Hospital 3	Validation Hospital 4
Chronic Heart Failure	r_{pb}	0.005	0.250	0.181	-0.038	0.007
	P value < 0.05	×	✓	✓	×	×
Ischaemic heart diseases	r_{pb}	0.056	-0.063	-0.016	0.015	0.015
	P value < 0.05	×	×	×	×	×
Pulmonary heart disease	r_{pb}	-0.058	0.164	0.013	-0.029	-0.052
	P value < 0.05	×	✓	×	×	×
Other forms of heart disease	r_{pb}	0.076	0.019	0.142	0.040	0.901
	P value < 0.05	×	×	✓	×	×

The calculation of the *P* value linked with the r_{pb} involves a statistical hypothesis test. The null hypothesis posits no correlation between the binary heart failure-related feature and mortality, while the alternative hypothesis suggests a correlation. Specifically, a t-test is employed, assuming that the mean mortality for the two groups defined by the binary heart failure-related feature is either the same (null hypothesis) or different (alternative hypothesis).

To assess and compare the association of heart failure-related features with ICU mortality against the association of the binray features used in our model with ICU mortality, we provide the relevant r_{pb} and the associated significance of the features used in our model in Supplementary Table S4. The results reveal that the majority of features exhibit high absolute values of r_{pb} across all hospitals, underscoring their substantial discriminative power in stratifying mortality.

Supplementary Table S4. The representation of r_{pb} for binary features used in our model and their correlation with patient mortality, categorized by hospital. A value of r_{pb} close to 0 suggests a weak association, while values close to 1 or -1 indicate a strong association. P values < 0.05 indicate significant difference in the mean mortality for the two groups defined by the binary features used in our model.

**Features in the Hybrid Modeling Framework**	**Significance**	**Derivation Hospital**	**Validation Hospital 1**	**Validation Hospital 2**	**Validation Hospital 3**	**Validation Hospital 4**
Liver Failure	r_{pb}	0.466	0.377	0.604	0.310	0.474
	P value < 0.05	✓	✓	✓	✓	✓
Renal failure	r_{pb}	0.349	0.293	0.562	0.405	0.353
	P value < 0.05	✓	✓	✓	✓	✓
Sepsis	r_{pb}	0.267	0.284	0.287	0.293	0.495
	P value < 0.05	✓	✓	✓	✓	✓
Diseases of liver	r_{pb}	0.455	0.314	0.548	0.267	0.475
	P value < 0.05	✓	✓	✓	✓	✓
Other bacterial diseases	r_{pb}	0.296	0.216	0.181	0.298	0.444
	P value < 0.05	✓	✓	✓	✓	✓
Diseases of the genitourinary system	r_{pb}	0.240	0.169	0.345	0.286	0.283
	P value < 0.05	✓	✓	✓	✓	✓
Mycoses	r_{pb}	0.349	0.222	0.106	0.277	0.363
	P value < 0.05	✓	✓	✓	✓	✓
Mental and behavioural disorders	r_{pb}	-0.335	-0.219	-0.284	-0.287	-0.180
	P value < 0.05	✓	✓	✓	✓	✓
Organic, including symptomatic, mental disorders	r_{pb}	-0.271	-0.194	-0.232	-0.234	-0.133
	P value < 0.05	✓	✓	✓	✓	✓
ARDS	r_{pb}	0.547	0.512	0.509	0.508	0.514
	P value < 0.05	✓	✓	✓	✓	✓
Polyneuropathies and disorders of the peripheral nervous system	r_{pb}	-0.144	-0.036	-0.359	0.155	0.159
	P value < 0.05	✓	×	✓	✓	✓
Respiratory diseases principally affecting the interstitium	r_{pb}	0.551	0.504	0.508	0.494	0.495
	P value < 0.05	✓	✓	✓	✓	✓

5 Decision process for selecting a suitable statistical test

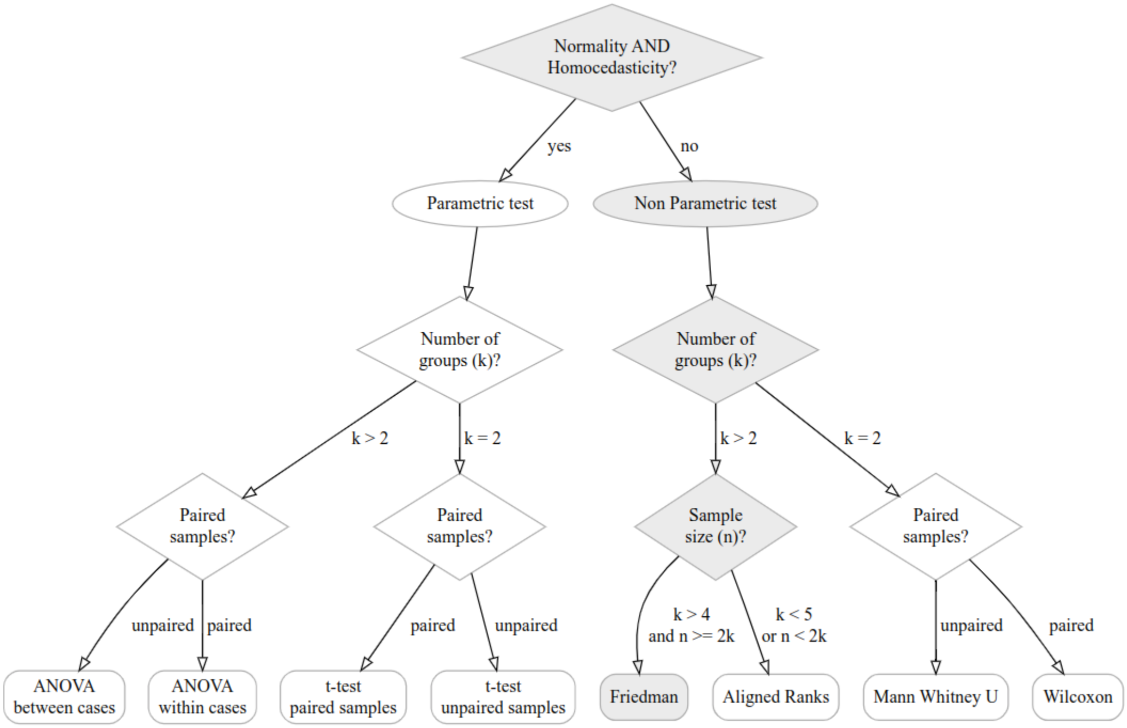

Supplementary Figure S4. The decision process for selecting the appropriate statistical test to quantitatively measure the consistency of the interpretations provided by SHAP values. The STAC library[3] was utilized to identify the optimal test. Due to the lack of normality and homoscedasticity in the SHAP value distributions, a non-parametric test was chosen. The Friedman test was identified as an appropriate choice for our analysis due to the necessity of comparing more than two distributions ($k = 5$). This test was particularly fitting, given that we utilized $n = 80$ ($\geq 2k$) validation data subsets in our examination.

6 Optimized hyperparameter of the XGBoost model

	**Derivation Hospital**	**Validation Hospital 1**	**Validation Hospital 2**	**Validation Hospital 3**	**Validation Hospital 4**
**Colsample by tree**	0.5	0.75	0.75	0.75	0.5
**Gamma**	0	0.25	1	1	0
**Learning rate**	0.05	0.01	0.1	0.1	0.05
**Max depth**	4	4	4	4	4
**Reg lambda**	0	1	1	1	1
**Subsample**	0.75	0.75	0.75	0.5	0.5

Supplementary Table S5. Optimized hyperparameters in grid cross-validation for the training of the XGBoost model.

References

1. Debray, T. P. *et al.* A new framework to enhance the interpretation of external validation studies of clinical prediction models. *J. clinical epidemiology* **68**, 279–289 (2015).

2. Vincent, J. L. *et al.* The sofa (sepsis-related organ failure assessment) score to describe organ dysfunction/failure: On behalf of the working group on sepsis-related problems of the european society of intensive care medicine (see contributors to the project in the appendix) (1996).

3. Rodríguez-Fdez, I., Canosa, A., Mucientes, M. & Bugarín, A. STAC: a web platform for the comparison of algorithms using statistical tests. In *Proceedings of the 2015 IEEE International Conference on Fuzzy Systems (FUZZ-IEEE)* (2015).
